# Liver glycogen phosphorylase is upregulated in glioblastoma and provides a metabolic vulnerability to high dose radiation

**DOI:** 10.1038/s41419-022-05005-2

**Published:** 2022-06-28

**Authors:** Christos E. Zois, Anne M. Hendriks, Syed Haider, Elisabete Pires, Esther Bridges, Dimitra Kalamida, Dimitrios Voukantsis, B. Christoffer Lagerholm, Rudolf S. N. Fehrmann, Wilfred F. A. den Dunnen, Andrei I. Tarasov, Otto Baba, John Morris, Francesca M. Buffa, James S. O. McCullagh, Mathilde Jalving, Adrian L. Harris

**Affiliations:** 1 Molecular Oncology Laboratories, Department of Oncology, Oxford University, MRC Weatherall Institute of Molecular Medicine, John Radcliffe Hospital, Oxford, UK; 2 Department of Medical Oncology, University Medical Centre Groningen, University of Groningen, Groningen, the Netherlands; 3 The Breast Cancer Now Toby Robins Research Centre, The Institute of Cancer Research, London, UK; 4 Department of Chemistry, University of Oxford, Oxford, UK; 5 Department of Oncology, Democritus University of Thrace, Alexandroupolis, Greece; 6 The Bioinformatics Hub, Department of Oncology, University of Oxford, Oxford, UK; 7 Wolfson Imaging Centre Oxford, MRC Weatherall Institute of Molecular Medicine, Oxford, UK; 8 Department of Pathology, University Medical Centre Groningen, University of Groningen, Groningen, the Netherlands; 9 Oxford Centre for Diabetes, Endocrinology and Metabolism, University of Oxford, Churchill Hospital, Oxford, UK; 10 School of Biomedical Sciences, Ulster University, Coleraine, Northern Ireland UK; 11 Tokushima University Graduate School, Tokushima, Japan; 12 Department of Physiology, Anatomy and Genetics, University of Oxford, Oxford, UK; 13 Department of Oncology, University of Oxford, Churchill Hospital, Oxford, UK

**Keywords:** Cancer metabolism, CNS cancer

## Abstract

Channelling of glucose via glycogen, known as the glycogen shunt, may play an important role in the metabolism of brain tumours, especially in hypoxic conditions. We aimed to dissect the role of glycogen degradation in glioblastoma (GBM) response to ionising radiation (IR). Knockdown of the glycogen phosphorylase liver isoform (PYGL), but not the brain isoform (PYGB), decreased clonogenic growth and survival of GBM cell lines and sensitised them to IR doses of 10–12 Gy. Two to five days after IR exposure of PYGL knockdown GBM cells, mitotic catastrophy and a giant multinucleated cell morphology with senescence-like phenotype developed. The basal levels of the lysosomal enzyme alpha-acid glucosidase (GAA), essential for autolysosomal glycogen degradation, and the lipidated forms of gamma-aminobutyric acid receptor-associated protein-like (GABARAPL1 and GABARAPL2) increased in shPYGL U87MG cells, suggesting a compensatory mechanism of glycogen degradation. In response to IR, dysregulation of autophagy was shown by accumulation of the p62 and the lipidated form of GABARAPL1 and GABARAPL2 in shPYGL U87MG cells. IR increased the mitochondrial mass and the colocalisation of mitochondria with lysosomes in shPYGL cells, thereby indicating reduced mitophagy. These changes coincided with increased phosphorylation of AMP-activated protein kinase and acetyl-CoA carboxylase 2, slower ATP generation in response to glucose loading and progressive loss of oxidative phosphorylation. The resulting metabolic deficiencies affected the availability of ATP required for mitosis, resulting in the mitotic catastrophy observed in shPYGL cells following IR. PYGL mRNA and protein levels were higher in human GBM than in normal human brain tissues and high PYGL mRNA expression in GBM correlated with poor patient survival. In conclusion, we show a major new role for glycogen metabolism in GBM cancer. Inhibition of glycogen degradation sensitises GBM cells to high-dose IR indicating that PYGL is a potential novel target for the treatment of GBMs.

## Introduction

Glioblastoma (GBM, WHO grade IV) is the most common and aggressive brain tumour. Despite standard treatment, comprising surgical resection and chemoradiotherapy, most patients relapse at the primary site. The disease is incurable with a median overall patient survival of only 15 months [1]. Novel treatments are urgently needed. GBM resistance to the current standard therapies is characterised by hypoxia, glioblastoma stem-like cells and metabolic reprogramming. Thus, targeting metabolism in order to increase sensitivity to anticancer therapies is of great interest.

In cancer cells, metabolic reprogramming provides energy, synthetic metabolites and maintains redox balance [2]. The best-known metabolic shift is the Warburg effect. It describes increased glucose uptake by cancer cells and high glycolysis rates even in the presence of sufficient oxygen [3]. Highly proliferative GBM cells have an increased pentose phosphate pathway (PPP) compared to migratory counterparts [4]. The PPP is required to provide nucleotides and utilises glucose-6-phosphate from glycolysis or glycogen degradation [5]. In vivo flux analyses using ^13^C-glucose in 11 patients with GBM demonstrated that less than 50% of the acetyl-CoA pool in GBM is generated from blood-borne glucose [6]. This indicates that other sources than glucose, such as glycogen, may contribute to tumour bioenergetics.

Glycogen is the intra-cellular storage form of glucose. Glycogen synthase (GYS) is the rate-limiting enzyme of glycogen synthesis [7, 8] and catalyses the chain formation from uridine diphospho-glucose (UDP-glucose) [8, 9] while branching is catalysed by glycogen branching enzyme (GBE) [7]. Glycogen degradation occurs both in the cytosol and in lysosomes. In the cytosol, glycogen is broken down by glycogen phosphorylase (PYG) and glycogen debranching enzyme (DBE) [7, 8]. PYG is the rate-limiting enzyme in the cytosol and has three isoforms; liver (PYGL), brain (PYGB) and muscle (PYGM) [8]. In lysosomes, glycogen is degraded by alpha-acid glycosidase (GAA) called glycogen autophagy or glycophagy [7].

Glycogen storage is inversely correlated with cancer cell growth and is upregulated in response to hypoxia both in vitro and in vivo in multiple cancer cell lines and tissues including GBM [8, 10]. The “glycogen shunt” describes the channelling of glucose via glycogen to produce glucose-6-phosphate, which ensures homeostasis of metabolic intermediates and proper timing of glucose utilisation. This process is likely important in GBM due to intermittent hypoxia resulting in fluctuating cellular glucose requirements [11, 12].

We previously found that acute PYGL knockdown and subsequent glycogen accumulation in the GBM cell line U87MG resulted in reduced flux through the PPP and an increase in the production of reactive oxygen species (ROS), contributing to premature senescence and markedly impaired tumorigenesis [10]. ROS production can also induce DNA damage and radiation sensitivity. PPP is required for generating ROS scavengers, therefore this pathway is important in the radioprotection of cancer cells [13]. We hypothesised that PYGL inhibition and the subsequent reduction in PPP flux would increase the radiosensitivity of GBM cells [14]. This study demonstrates that inhibition of glycogen degradation by PYGL knockdown sensitises GBM cells to high dose IR through induction of metabolic stress, defective autophagy and mitophagy, and consequent mitotic catastrophy.

## Materials and Methods

### Cell lines and culture conditions

Glioblastoma cell lines (U87MG, T98G, U118MG, U251MG, LN18, LN229) were obtained from American Type Culture Collection (ATCC). Cell line authentication was carried out by STR analyses by Eurofins Genomics. The relevant characteristics of these cell lines are shown in Table S1. Cell lines were routinely grown in flasks (Corning) under standard conditions in a 5% CO_2_ incubator at 37 ^o^C in Dulbecco’s modified Eagle’s medium (DMEM) containing 10% Fetal bovine serum (FBS). All normoxia experiments were conducted in DMEM containing 5 mM glucose, unless otherwise indicated. All hypoxia experiments were conducted in DMEM containing 10 mM glucose. Spheroids were cultured in DMEM containing 25 mM glucose.

### Stable knockdown cell lines

Lentiviral transduction particles containing a PYGL short hairpin RNA (shRNA) expression cassette (5-CCGGTACCAGCTTGGATTGGATATACTCGAGTATATCCAATCCAAGCTGGTATTTTTG-3, TRCN0000296097) or a non-targeting shRNA sequence (5-CCGGCAACAAGATGAAGAGCACCAACTCGAGTTGGTGCTCTTCATCTTGTTGTTTTT-3, SHC0002V) were purchased from Sigma-Aldrich. GBM cell lines U87MG, U251MG, T98G and LN229, were transduced with a multiplicity of infection of 3, in the presence of 6 μg/ml Polybrene (Sigma). Cells expressing the shRNA were selected in puromycin (Invitrogen)-containing medium (1 μg/ml). The knockdown effectiveness was assessed by immunoblotting.

### Proliferation assays and siRNA experiments

Reverse transfection was carried out using RNAiMax reagent (Life Technologies). Cells were transfected with 20 nM of ON-TARGET plus SMART pool siRNA for non-targeting Pool (D-001810-10-05), PYGL (L-009569-00-0005) and PYGB (L-009587-01-0005). For cell proliferation experiments (*n* = 3), cells were transfected at a density of 10^5^ cells per well (U87MG, U118MG, U251MG, T98G, LN18) and 50.000 cells per well (LN229) in a 6-well plate and counted 5 days later using the cellometer auto T4 (Nexcelom Bioscience).

### Spheroid generation and growth after ionizing radiation (IR)

Spheroids (*n* = 8 per group) were generated using 2.5 × 10^4^ cells in 200 μl cell suspension. Spheroids were added to each well of a 96-well plate, with a round and Ultra Low attachment bottom (Corning Incorporated), and aggregated by centrifugation at 400 g for 10 min. Two days later, the spheroids were irradiated with a single dose of 12 Gy and subsequently monitored every 3 days for 3 weeks. Pictures were taken with the EVOS XL Core microscope (Advanced Microscopy Group, Waltham, MA, USA) and analysed using SpheroidSizer software [15].

### Antibodies used for cell line experiments

The antibodies used were: rabbit polyclonal anti-PYGL (1:1000, HPA000962, Sigma-Aldrich), rabbit polyclonal anti-PYGB (1:1000, HPA031067, Sigma-Aldrich), rabbit polyclonal anti-GBE1 (1:1000, HPA038073, Sigma-Aldrich), rabbit monoclonal anti-GYS1 (1:1000, ab40810, Abcam), rabbit polyclonal anti-phospho-glycogen synthase Ser641 (1:1000, 3891, Cell Signaling), rabbit polyclonal anti- phosphorylated replication protein A subunit32 (pRPA32 S4/S8, 1:1000, A300-245A, Bethyl), mouse monoclonal anti-phospho S139 H2A.X JBW301 (1:1000, 05-636, Millipore), mouse anti-p21 (1:1000, 556430, BD pharma), rabbit polyclonal anti microtubule associated protein 1 light chain 3 alpha (MAP1LC3A) (1:1000, ab62720, Abcam, a synthetic peptide PSDRPFKQRRSFADR conjugated to KLH by a Cysteine residue linker, corresponding to amino acids 2-15 of Human MAP1LC3A), mouse monoclonal anti microtubule associated protein 1 light chain 3 beta (MAP1LC3B) (1:1000, 5F10 Nanotools), rabbit polyclonal anti gamma-aminobutyric acid receptor-associated protein (GABARAP) (1:1000, PM037, MBL life science), rabbit polyclonal anti-GABARAP-like 1 (GABARAPL1) (1:1000, 11010-1-AP, Proteintech), rabbit polyclonal anti-GABARAPL2 (1:1000, 18724-1-AP, Proteintech), mouse monoclonal anti-GAA (1:1000, sc-373745, Santa Cruz), rabbit polyclonal anti β-galactosidase (1:1000, 15518-1-AP, Proteintech), rabbit monoclonal anti-pAMPK T172 (1:1000, 2535, Cell Signaling) and rabbit polyclonal anti-phospho-Acetyl-CoA Carboxylase (Ser79) (1:1000, 3661, Cell Signaling).

### Western blotting procedures

Cells were washed with cold phosphate-buffered saline (PBS) and then lysed in radioimmunoprecipitation assay (RIPA) buffer (R0278, Sigma-Aldrich) containing protease (cOmplete^™^, Mini, EDTA-free Protease Inhibitor Cocktail) and phosphatase (PhosSTOP^™^ inhibitor cocktail, Roche) inhibitors. Total protein concentrations were estimated using the DC Protein Assay kit (Bio-Rad). Per well, 15 μg protein was loaded on NuPAGE 12.5% Bis-Tris (for MAP1LC3 and GABARAP family proteins*)* or NuPAGE gradient 4-12% Bis-Tris gels (Invitrogen) and transferred on PVDF membranes (PVDF-PSQ, pore size 0.22um, for MAP1LC3 and GABARAP family proteins and PVDF, pore size 0.45 µm, for all other proteins, Millipore Corp) overnight at 20 V.

To minimise the non-specific binding sites, membranes were incubated for 2 h at room temperature (RT) with 5% non-fat dry milk in 150 mM NaCl, 10 mM Tris, 0.1% Tween-20 pH 7.5 (TBS-T). After, the membranes were hybridised overnight at 4 °C with primary antibodies. The membranes were then hybridised for 2 h at RT with the secondary antibody, goat polyclonal to rabbit IgG (1:2000, Dako) or rabbit polyclonal to mouse IgG (1:2000, Dako) conjugated to horseradish peroxidase. ECL Prime (GE Healthcare, Chalfont St Giles, UK) was used to detect membrane immunoreactivity. Visualisation was performed using an ImageQuant LAS4000 (GE Healthcare). Each blot was subsequently stripped incubated in 2% SDS (w/v), 62.5 mM Tris-HCl (pH 6.8), 100 mM β-mercaptoethanol for 30 min at 60 °C, rinsed twice for 10 min each with TBS-T, dried overnight, re-hybridised with a monoclonal antibody to β-actin conjugated to horseradish peroxidase (1:20000, 3854, Sigma) and developed as described.

### Glycogen assay

In 6 GBM cell lines, we analysed glycogen levels in response to 24 h incubation in hypoxia (n = 3 per group). Hypoxia experiments were carried out at 0.1% O_2_ / 5% CO_2_ in N_2_ using an Invivo2 400 workstation (Ruskinn Technology Ltd, Bridgend, UK). Briefly, 7 × 10^5^ cells were seeded in 10 cm^2^ dishes (10 mM glucose DMEM, 10% FBS). The next day, these cells were exposed to hypoxic conditions for 24 h. Glycogen levels were measured using a Glycogen Assay Kit (K646, BioVision) following manufacturer’s instructions. Cells were homogenised with 200 μl of dH_2_O on ice and then boiled for 10 min. Homogenates were spun at 18,000 g for 10 min and supernatants were assayed for glycogen content. Results were normalised by number of cells.

### Ionising radiation (IR)

Incucyte was used to track cell proliferation over time in response to IR. Control (shControl) and shPYGL U87MG cells were seeded at a density of 20,000 per well in 6-well plates and the next day exposed to single IR doses of 4, 6, 8, 10 and 12 Gy (IBL637, Caesium-137, dose rate 0.054 Gy/sec). Also, daily fractionated schedules of 3 × 6 Gy and 2 × 8 Gy, and weekly fractionated doses of 2 × 12 Gy were used. At the end of the experiments the plates were stained using Coomassie blue. For immunoblotting and immunofluorescence, cells were seeded overnight and irradiated with 12 Gy the next day and harvested at the corresponding time points used for analysis.

### Metabolic flux assay

The mitochondria oxygen consumption rate (OCR, O_2_ mpH/min) and extracellular acidification rate (ECAR, mpH/min) of the shControl and shPYGL cells were analysed using an XF^e^96 extracellular flux analyzer (Seahorse Bioscience). Cells were seeded in a Seahorse XF96 well plate (Seahorse Bioscience) and incubated in 5 mM glucose DMEM medium, 10% FBS at 37 °C with 5% CO_2_. The OCR and ECAR of these cells were analysed 48 h (14,000 cells were seeded for this time point) and 5 days (7,000 cells were seeded for this time point) after IR. For the OCR assay, all wells were washed twice with 200 μl of XF DMEM medium (5 mM glucose, 1 mM pyruvate, 2 mM L-glutamine, pH 7.4). Afterwards, a final volume of 175 μl of XF medium was added to each well. Subsequently, the plates were incubated for 1 h at 37 °C in a non-CO_2_ incubator before the start of the assay. OCR was analysed in four stages: basal respiration, mitochondrial complex V inhibition (1 μM oligomycin), maximal respiration (0.5 μM carbonyl cyanide 4-(trifluoromethoxy)phenylhydrazone, FCCP), and electron transportation chain inhibition (0.5 μM rotenone and 0.5 μM antimycin A). For ECAR analysis, cells were washed twice with 200 μl of XF DMEM medium (0 mM glucose, 1 mM pyruvate, 2 mM L-glutamine, pH 7.4) and a final volume of 175 μl of XF medium was added to each well. Subsequently, the plates were incubated for 1 h at 37 °C in a non-CO_2_ incubator before start of the assay. ECAR was analysed in 3 stages: basal (0 mM glucose), glycolysis induction (10 mM glucose) and maximal glycolysis induction (0.5 μM rotenone and 0.5 μM antimycin A).

### Epifluorescence imaging of ATP/ADP ratio in cell lines

Real-time cytosolic ATP/ADP ratio in cell lines was reported by a recombinant fluorescent sensor, derived by fusion of a yellow fluorescent protein (YFP) variant with the bacterial regulatory protein GlnK1 [16]. This methodology allows the signal to be acquired from multiple populations of cells simultaneously, at a single-cell resolution. The sensor was delivered into the cells via an adenoviral vector at ca. 10^4^ infectious units per cell 24 h prior to the experiment, as described earlier [17]. The imaging experiments were performed on a Carl Zeiss AxioZoom.v16 microscope using a 2.3×/0.56 objective. Cell lines expressing the sensor were cultured on a coverslip (thickness 1.5 micron, 0.17 mm), which was then positioned inside an imaging chamber [18] and continuously perfused with bath solution at 37 °C containing various stimuli, as indicated. The bath solution contained, mM: 140 NaCl, 4.6 KCl, 2.6 CaCl_2_, 1.2 MgCl_2_, 1 NaH_2_PO_4_, 5 NaHCO_3_, 10 HEPES, (pH 7.4, with NaOH). The fluorophore was excited at 490 nM and the emission was collected at 535 nM. The images were acquired every 30 seconds.

### Confocal microscopy

For confocal microscopy, cells were cultured on coverslips, rinsed in PBS and fixed in 4% (v/v) paraformaldehyde in PBS. Subsequently, the samples were permeabilised using 0.2% (v/v) Triton X-100 in PBS, blocked in 3% (w/v) bovine serum albumin in PBS containing 0.1% Tween-20 (PBST) and incubated with a primary antibody diluted in blocking buffer. For secondary detection, goat anti-mouse/rabbit IgG labelled with Alexa Fluor 488/594 (Life Technologies) was used. DNA was stained using 1 μM DAPI (4′,6-diamidino-2-phenylindole). Coverslips were mounted using ProLong Gold antifade (Life Technologies). Confocal images were captured on a Zeiss 510 inverted confocal microscope using a Plan Apochromat 63 × 1.40NA oil immersion objective lens (Carl Zeiss GmbH, Jena, Germany).

### Live Immunofluorescence imaging

To analyse cell division in response to IR, we performed live imaging using SiR-DNA (SiR-Hoechst, Spirochrome AG, Switzerland), which is based on the DNA minor groove binder bisbenzimide and labels DNA in live cells with high specificity and low background [19]. To assess phototoxicity caused by time-lapse imaging, separate wells in the imaging plate were exposed to fluorescence excitation light only at 0 and 24 h time points. For live confocal imaging, 4000 cells were seeded overnight in µ-Slide with 8 wells with a glass-bottom (ibidi, 80827). The next day, the shControl and shPYGL cells were irradiated and live imaged at time points 72–96h post IR. Live imaging was conducted every 2 min using a confocal spinning disc (Zeiss Cell Observer Spinning Disc Confocal), plan Apochromat 20 × 0.8NA air objective or Plan Apochromat 63 × 1.40NA oil immersion objective, a Hamamatsu ORCA Flash 4 v2 CMOS camera, equipped with lasers at respectively 405, 488, 561, and 640 nm along with appropriate fluorescence emission bandpass filters.

To analyse the mitochondrial phenotype we used the Mitotracker Green FM (Molecular Probes, M7514). Cells were incubated for 30 min in 100 nM Mitotracker Green prior to imaging. In parallel we used LysoTracker® Red DND-99 (Molecular Probes, L7528) to label cellular acidic vesicular organelles such as lysosomes. Cells were incubated for 30 min in Lysotracker Red prior to imaging.

For the senescence staining, we used SPiDER-βGal from Dojindo Molecular Technologies, Inc. (Rockville, MD, USA). SPiDER-βGal (1 μM) was added to the culture medium and incubated for 30 min. Fluorescence microscopy was performed with the following filters: excitation wavelength range 450–490 nm and emission wavelength range 500–550 nm. Transmitted light differential interference contrast images were obtained at the same time.

### Gene expression analyses on data of patients with GBM

Publicly available raw microarray gene expression data from brain tumours, normal and diseased brain tissue, and post-mortem brain tissue (Table S2) were collected from the Gene Expression Omnibus (GEO) [20]. Analysis was confined to samples hybridised to the Affymetrix HG-U133 Plus 2.0 (GEO accession number GPL570) platform. The robust multi-array average algorithm was used for pre-processing, and aggregation of raw data and quality control of the resulting expression data was performed as previously described [21–23]. In gene expression profiling, the net measured expression level of an individual gene is the result of the integrated activity of latent underlying regulatory factors (*e.g*. experimental, genetic or non-genetic factors). The number and nature of these underlying regulatory factors and their effects on gene expression levels were determined by performing consensus independent component analysis (consensus-ICA) on these samples. Consensus-ICA defined so called transcriptional components (TCs) that represent the underlying regulatory factors. Within a TC, each individual gene has a specific weight that indicates to what extent and in which direction the underlying transcriptional regulatory factor influences the expression level of that gene. In addition to TCs, a so-called mixing matrix (MM) containing weights was also provided by consensus-ICA. In the MM, each column corresponds to a TC and each row corresponds to a sample. Each weight in the MM represents the ‘activity’ of the corresponding TC in an individual sample, called the activity score. With the resulting TCs we performed gene set enrichment analysis (GSEA) utilising 16 gene set collections (Table S3). We studied the top 10 enriched gene sets with a Z-score ≥3 within the TCs of interest (Tables S4-S6). The Cancer Genome Atlas Program (TCGA) GBM data (profiled using microarrays) was downloaded from TCGA DCC (GDAC), release 2014-10-17. This dataset contained 525 tumour samples with annotation data. Valid survival data was available for 518 of these tumour samples, which were used for the analyses.

### Immunohistochemistry (IHC)

Full-slides containing normal brain and GBM tissue were stained for glycogen, GYS1, PYGL, PYGB and glucose transporter 1 (GLUT-1) expression. The tissue microarray (TMA) contained tissue samples of adult patients diagnosed with GBM (World Health Organization Grade IV astrocytoma) from November 2005 till February 2014 at the University Medical Centre Groningen, the Netherlands. The CONSORT diagram describing patient selection is shown in Fig. S5A. Institutional review board approval for this study was obtained and need for informed consent was waived (Medical Ethical Committee Number University Medical Center Groningen, the Netherlands: 2018/416). Cellular, non-hypoxic tumour areas, as judged microscopically after standard haematoxylin and eosin staining by a neuropathologist (WdD), were cored from formalin-fixed paraffin-embedded GBM tissue blocks. The TMA contained 4 tissue cores per patient. The following primary antibodies were used: mouse anti-Glycogen (gift from Professor O. Baba [24]), GYS1 (ab40810, Abcam), PYGL (HPA000962, Sigma-Aldrich), PYGB (HPA031067, Sigma-Aldrich), GLUT-1 (ab115730, Abcam). The IHC protocol used is described in the supplemental methods (Immunohistochemistry).

Aperio ImageScope software (version 12.3.2.8013, Leica Biosystems) was used for hand-annotating tissue cores containing at least 25% evaluable vital tumour tissue by one author (AMH). Only patients of whom at least 3 evaluable tissue cores were available were included in the analyses. Areas without tumour cells, such as necrosis, large blood vessels, collagen, and artifacts were excluded from the annotated areas. The annotation was supervised by a neuropathologist (WdD). Two authors (AMH, WdD) set the Aperio Positive Pixel Count v9 Algorithm parameters to exclude all non-specific background signals and define positive and strongly positive pixels.

An H-score was calculated for each annotated tissue according to the following formula: score = (1 x number of positive pixels + 2 x number of strongly positive pixels)/area of annotation. There was a strong correlation between manually and automatically derived H-scores using Aperio’s positive pixel count algorithm (supplemental methods).

For each patient, the mean score of all evaluable tissue cores was determined per protein staining. The distribution of mean scores was right-skewed for all protein stainings except for PYGB (Fig. S6). The medians of these distributions were used as cut-off values to determine low and high expression groups for each staining. Based on those cut-off values, Kaplan-Meier curves were plotted for patients younger than 70 years of age at surgery. The cut-off value of 70 years of age was applied since this is a recognised prognostic factor for patients with GBM.

### Statistical analysis

All cell line statistical analyses were carried out using GraphPad Prism (v. 6.0) by unpaired t-test or one-way/two-way ANOVA followed by Tukey’s multiple comparisons test on independent experimental replicates, unless otherwise indicated. The epifluorescence imaging data of ATP/ADP ratio in cell lines was analysed using FIJI (ImageJ). Briefly, image sequences were registered using TurboReg plug-in [25] and the regions of interest corresponding to single cells were defined based on the intensity maxima. The intensity time course was numerically analysed using IgorPro (Wavemetrics). Statistical analysis was performed using R [26]. Data is presented as the mean values ± standard error of the mean (S.E.M) unless otherwise specified. Comparisons within one experiment were performed using Kruskal-Wallis test with Nemenyi post-hoc analysis (independent samples) or Friedman test with Nemenyi post-hoc analysis (dependent samples). Differences with *P* < 0.05 were considered statistically significant. For TCGA data analyses, Cox proportional hazard model was fit to median dichotomised mRNA abundance data for genes included in survival analysis and *P* values were estimated using Wald test. Analyses of histological protein expression levels were conducted using IBM SPSS Statistics 23 by Spearman’s correlation and Kaplan-Meier curves were compared with log-rank tests.

## Results

### Downregulation of PYGL decreased cell growth in GBM cell lines

**Fig. 1:**
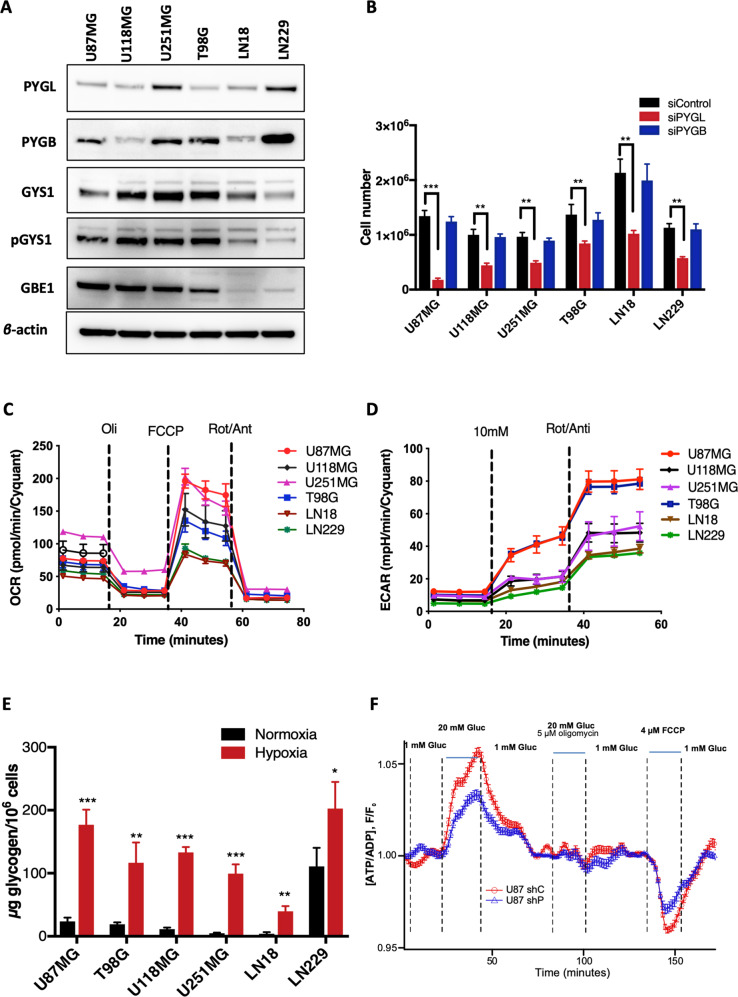
Characterisation of expression levels of glycogen related enzymes, effect of PYGL knockdown, glycogen accumulation and bioenergetics of six glioblastoma (GBM) cell lines. **A** Differential expression of glycogen related proteins in GBM cell lines: glycogen phosphorylase liver isoform (PYGL), glycogen phosphorylase brain isoform (PYGB), glycogen synthase 1 (GYS1), phospho-glycogen synthase (pGYS1), glycogen branching enzyme 1 (GBE1) and glycogen debranching enzyme (GDE). Beta-actin was used as loading control. **B** Effects of PYGL knockdown (siPYGL) and PYGB knockdown (siPYGB) on cell numbers after 5 days in six GBM cell lines (*n* = 3, error bars are ± SD, ***p < 0.001, repeated measures ANOVA, Tuckey post hoc). **C** Oxygen consumption rate (OCR) was measured in six GBM cell lines, by a mitochondria stress test using oligomycin (an ATP synthase inhibitor), FCCP (an electron transport chain uncoupler) and rotenone/antimycin (inhibitors of electron transport chain). **D** Extracellular acidification rate (ECAR) was measured in six GBM cell lines. Cells incubated in 0 mM glucose for 1 h in CO_2_ free conditions were exposed to 10 mM glucose and rotenone/antimycin. **E** Glycogen levels in response to hypoxia in GBM cancer cell lines. Cell lines were incubated in 21% (normoxia) and 0.1% (hypoxia) oxygen for 24 h. Glycogen levels were analysed using a colorimetric kit (*n* = 3, Error bars are ± SD, *p < 0.05, **p < 0.01, ***p < 0.001, repeated measures ANOVA, Tukey post hoc). **F** Kinetics of the cytosolic ATP/ADP ratio [ATP/ADP]_cyt_ in response to high (20 mM) glucose in control (shControl) U87 cells (red) and PYGL knockdown (shPYGL) U87 cells (blue).

Analysis of the basal protein levels of enzymes related to glycogen metabolism in six GBM cell lines showed variable expression of PYGL, PYGB, GYS1 and GBE1 (Fig. 1A). PYGL was present in all cell lines, with highest protein levels in U251MG and LN229 cell lines. GBE1 was lowest in LN18 and LN229. GYS1 levels were lower in LN229 cells compared to the other GBM cell lines. Expression levels of PYGL, examined using Gene Expression Profiling Interactive Analysis [27], was higher in human GBM tissue compared to normal human brain tissue while for PYGB there was no difference (Fig. S1A). PYGM was downregulated in GBM cancer compared to normal brain tissue (Fig. S1A).

To further investigate the role of PYGL in GBM cells, we downregulated PYGL and PYGB using siRNA (Fig. S1B). PYGL downregulation decreased cell numbers on day 5 in all GBM cell lines while PYGB knockdown had no effect (Fig. 1B).

### Basal bioenergetic profiles and glycogen content in GBM cell lines are heterogeneous

Mitochondrial function was determined by Seahorse analysis. In basal conditions, U251MG cells had the highest basal OCR of the six GBM cell lines (Figs. 1C, S2A), while after mitochondrial uncoupling of OXPHOS to induce maximal respiration, U87MG and U251MG cells had the highest OCRs (Figs. 1C, S2B). U87MG and T98G had the highest ECAR, of the six GBM cell lines, both after glucose injection and after maximal stimulation (Figs. 1D, S2C, D).

The six GBM cell lines had a differential profile of basal glycogen levels in normoxia, with the highest glycogen level in the LN229 cells (Fig. 1E). In response to 24 h hypoxia (0.1% O_2_) glycogen content increased in all cell lines (Fig. 1E). Fold induction of glycogen after hypoxia varied from 2 to 17-fold (Fig. S2E). The cell lines with the highest glycolytic rates and high GBE1 levels, namely U87MG, T98G, U251MG and U118, had greater induction of glycogen levels after hypoxia than the LN229 cell line with the lowest ECAR and GBE1 levels (Figs. 1A, D, E and S2E). Despite the large fold induction of glycogen levels in LN18 cells (low ECAR and low GBE1) in response to hypoxia, the absolute values remained very low compared to the other cell lines.

We imaged the kinetics of the cytosolic ATP/ADP ratio ([ATP/ADP]_cyt_) in shControl and shPYGL U87MG cells, in response to the changes in extracellular glucose concentrations. In both shControl and shPYGL cells, an increasing in glucose concentration from 1 to 20 mM led to a rapid increase in [ATP/ADP]_cyt_, whereas the removal of the fuel resulted in the reversal of the effect (Fig. 1F). Notably, the glucose-induced increase of [ATP/ADP]cyt was substantially greater in shControl U87MG cells than in their shPYGL U87MG counterparts (Fig. 1F).

### Inhibition of PYGL sensitised GBM cells to IR

**Fig. 2:**
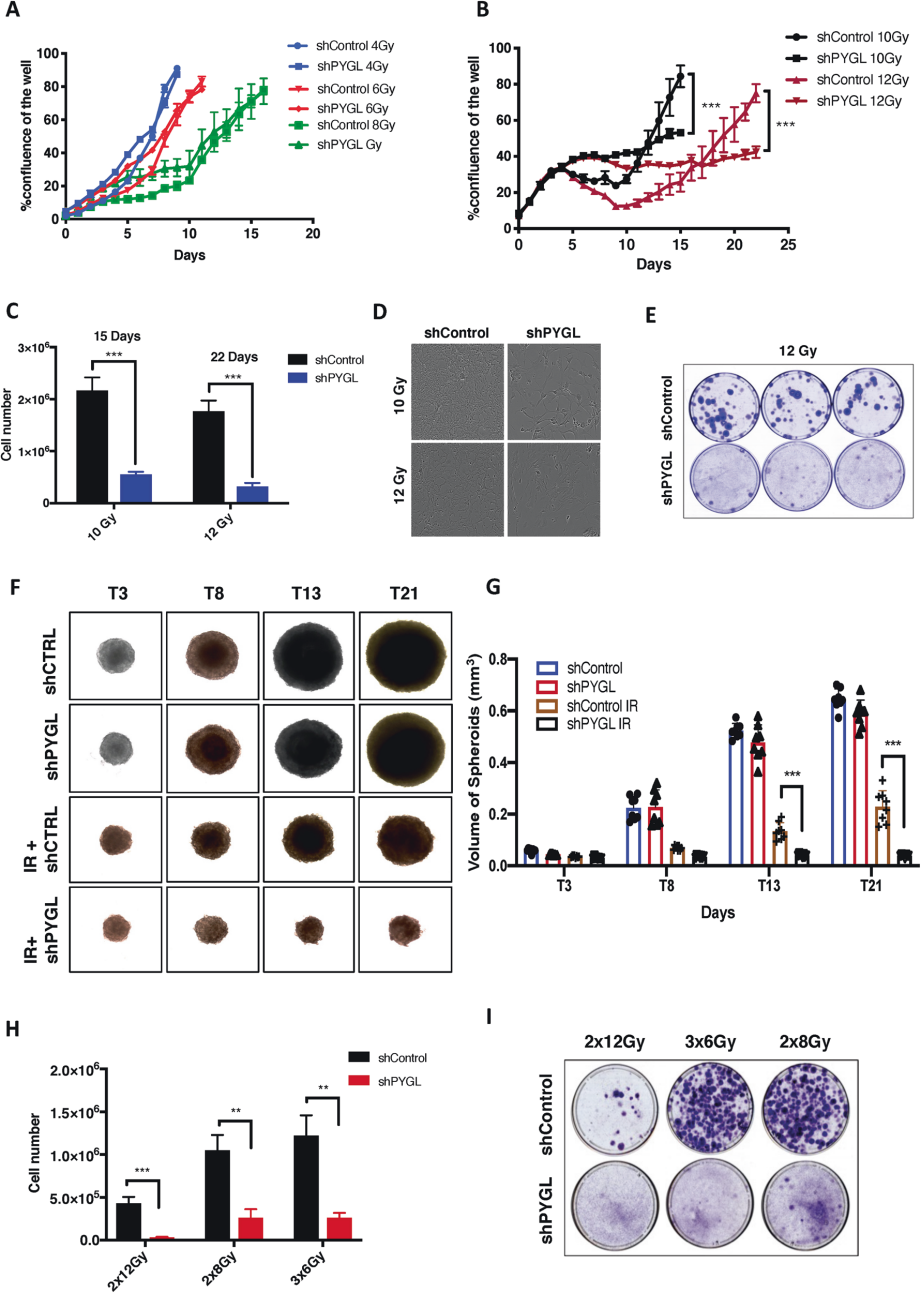
Inhibition of glycogen phosphorylase liver isoform (PYGL) sensitises glioblastoma (GBM) cells to ionising radiation (IR). **A**, **B** Cell proliferation in response to different doses of IR (*n* = 3, error bars are ± SD, ***p < 0.001). **C**, **D** Cell numbers and phase contrast images in response to 10 and 12 Gy IR in control (shControl) and PYGL knockdown (shPYGL) U87MG cells (*n* = 3, error bars are ±SD, ***p < 0.001, *p*-values calculated by unpaired t-test). **E** Coomassie blue staining was conducted 21 days after a single IR dose of 12 Gy in shControl and shPYGL U87MG cells. (*n* = 3). **F**, **G** Representative spheroid images **F** and quantification of spheroid volume (G) on day 3 (T3), day 8 (T8), day 13 (T13) and day 21 (T21) of shControl and shPYGL U87MG cells with or without IR on day 0 (*n* = 3, error bars are ±SD, ***p < 0.001, p values were calculated by Two Way ANOVA - Tuckey post-hoc). **H**, **I**. Cell numbers and Coomassie blue staining 21 days after different fractionated schedules of IR (2x12Gy, 3x6Gy, 2x8Gy) in shControl and shPYGL U87MG cells (n = 3, error bars are ±SD, **p < 0.01, ***p < 0.001, *p* values were calculated by unpaired t-test).

We investigated whether the shPYGL U87MG cells were more sensitive to different doses IR than shControl cells. After doses of 4, 6 and 8 Gy, both shPYGL and shControl U87MG cells showed similar growth patterns (Fig. 2A). In contrast, the shPYGL cell line was more sensitive to 10 Gy and 12 Gy than the shControl cell line, with lower cell counts and clonogenic survival at 15 and 22 days post IR (Fig. 2B-E). During the first 5 days after IR exposure of 10 Gy and 12 Gy, shPYGL and shControl U87MG cells showed a similar growth pattern (Fig. 2B). The shControl cells had a dip in cell growth between days 5 and 10 and recovered at 10 days after IR, while the shPYGL U87MG cells did not recover (Fig. 2B). The same effect of radiosensitisation by PYGL knockdown at higher IR doses occurred in U251MG and T98G (Fig. S3A, B). Spheroid growth of shPYGL cells was also impaired after high dose IR when compared to shControl U87MG cells (Fig. 2F, G). Higher doses of IR, such as 10 and 12 Gy, appear to define a critical threshold for differential response between the shPYGL and shControl U87MG cells. The same differential sensitivity was achieved using fractionated IR doses of 3 × 6 Gy, 2 × 8 Gy and 2 × 12 Gy as frequently used in the clinic (Fig. 2H, I).

### IR induced mitotic catastrophe after downregulation of PYGL

**Fig. 3:**
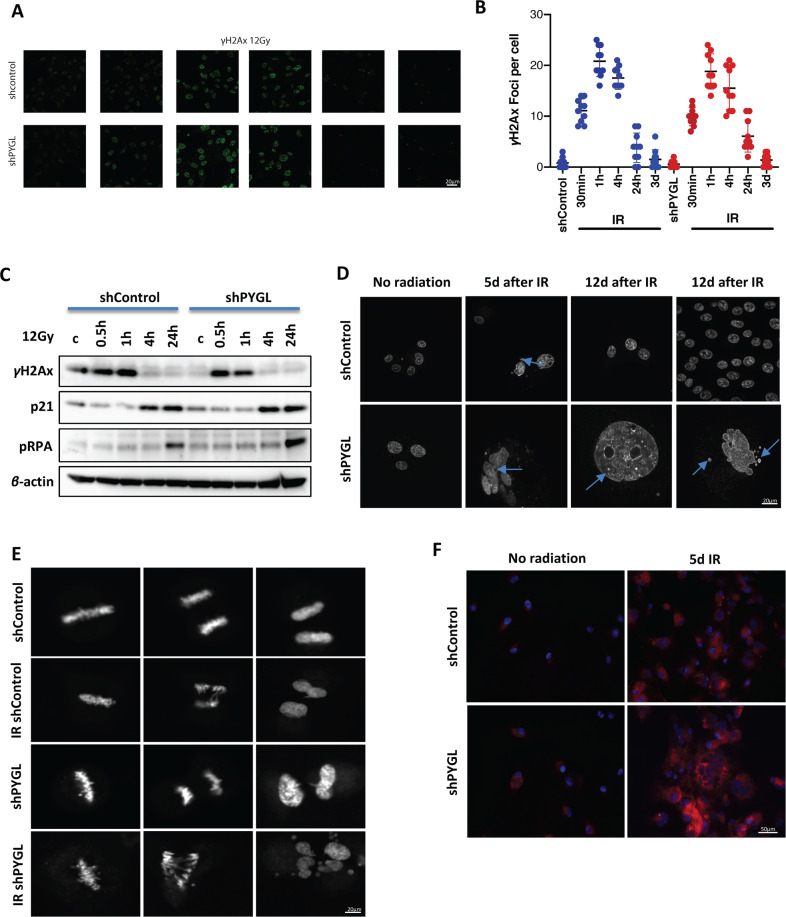
Ionising radiation (IR) induced mitotic catastrophe after downregulation of glycogen phosphorylase liver isoform (PYGL). **A**, **B** Immunofluorescence analysis (A) and quantitative analysis of γH2AX foci (B) following IR in control (shControl) and PYGL knockdown (shPYGL) U87MG cells. **C** Western blot analysis of γH2AX, p21, pRPA in response to IR in shControl and shPYGL U87MG cells. **D** Inhibition of PYGL induces mitotic catastrophe, DNA fragmentation and a giant cell phenotype following IR (blue arrows). Scale bars 20 μm. **E** Representative still images of a time relapse video 72-96 h after IR showing mitotic catastrophe during cell division in PYGL knockdown cells after IR. Scale bars 20 μm. **F** Representative senescence staining following IR in shControl and PYGLkd U87MG cells. SPiDER-βGal (1 μM) and fluorescence live microscopy was performed (excitation 450–490 nm, emission 500–550 nm) using the confocal spinning disc. Scale bars 20 μm.

The *γ*H2Ax foci formation, as a marker of DNA damage, did not differ in the size, number or time course following 12 Gy in shControl and shPYGL cells lines (Fig. 3A, B). We also investigated the role of PYGL inhibition on DNA damage and repair by immunoblot analysis of *γ*H2Ax, p21 and pRPA protein levels in shControl and shPYGL U87MG cells in response to 12 Gy IR (Fig. 3C). The induction of *γ*H2Ax (at 30 min and 1 h) and p21 (at 4 h and 24 h) protein levels after IR was similar in both the shControl and shPYGL U87MG cells. Also, there was similar induction of the DNA repair protein pRPA expression in shControl and shPYGL cells 24 h after IR. Thus, no differences in the induction of DNA damage and repair proteins after IR were detected between shControl and shPYGL U87MG cells.

Cell morphology revealed signs of mitotic catastrophe and the formation of giant polynucleated cells at 5 days post-IR, in both the shPYGL cells and the shControl cells (Fig. 3D). However, at that point there were also a few shControl cells that were -intact and without any signs of mitotic catastrophe or nuclear fragmentation. These cells were able to repopulate resulting in mainly normal-looking cells in the shControl 12 days after IR (Fig. 3D).

Live cell imaging using the Sir-DNA probe was used to investigate cell division after IR in shControl and shPYGL U87MG cells. 48 h to 5 days following IR, shPYGL cells failed to divide properly and showed signs of mitotic catastrophe (stills from video in Fig. 3E, complete video S4A). We further analysed whether a senescence-like phenotype was present in the giant multinucleated cells developed in shPYGL cells following IR. Indeed, β-gal staining was more evident in shPYGL at 5 days after IR (Fig. 3F).

### Downregulation of PYGL induced dysfunction of autophagy and altered the morphology of mitochondria following IR

**Fig. 4:**
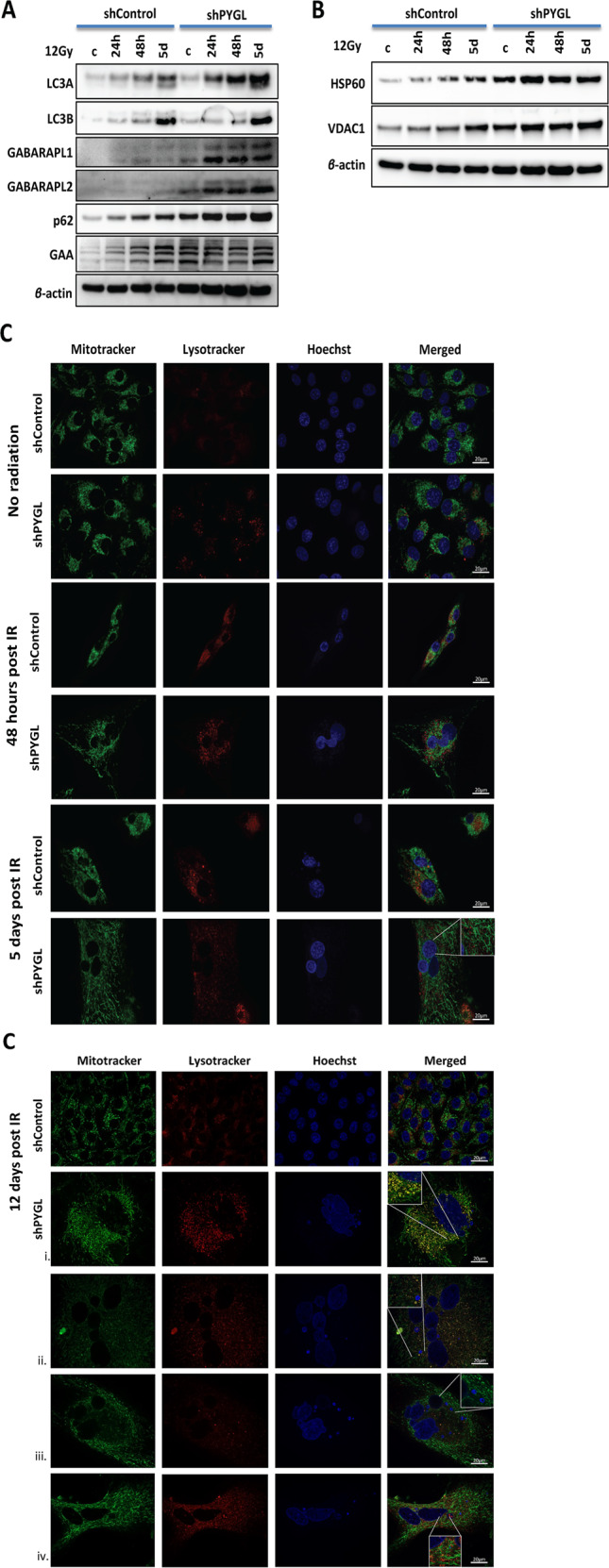
Inhibition of glycogen phosphorylase liver isoform (PYGL) induces dysfunction of autophagy and alters the morphology of mitochondria following ionising radiation (IR). **A** Western blot analysis of autophagy-related markers (microtubule associated protein 1 light chain 3 alpha (MAP1LC3A), microtubule associated protein 1 light chain 3 beta (MAP1LC3B), gamma-aminobutyric acid receptor-associated protein-like 1 (GABARAPL1), GABARAPL2 and p62 and the glycophagy-related marker alpha-acid glucosidase (GAA) 24 h, 48 h and 5 days after IR in control (shControl) and PYGL knockdown (shPYGL) U87MG cells. **B** Western blot analysis of mitochondria markers voltage-dependent anion-selective channel 1 (VDAC1) and heatshock protein 60 (HSP60) 24 h, 48 h and 5 days after IR in shControl and shPYGL U87MG cancer cells. **C** Immunofluorescence analysis of acid vesicles like lysosomes (lysotracker red) and mitochondria (mitotracker green) 48 h, 5 days and 12 days after IR in shControl and shPYGL U87MG cells. Elongation of mitochondria was seen five days after IR in shPYGL cells, while few cells from the shControl group remained intact. Twelve days after IR, shPYGL cells showed signs of mitotic catastrophe, fragmented and elongated mitochondria, and blocked mitophagy (strong colocalization with mitotracker green and lysotracker red) while the shControl relapse and regrow. Scale bars 20 μm.

Levels of autophagy proteins GABARAPL1 and GABARAPL2, p62 and of GAA (lysosomal glycogen degradation) were all increased in shPYGL cells compared to control U87MG cells (Fig. 4A). This is a potential compensatory mechanism for glycogen degradation. Following IR, the lipidated forms of the GABARAPL1 (GABARAPL1-II), GABARAPL2 (GABARAPL2-II) and MAP1LC3A (MAP1LC3A-II) accumulated over time in shPYGL cells compared to shControl U87MG cells after IR (Fig. 4A). The p62 accumulation together with increased levels of the lipidated form of the MAP1LC3 and GABARAP family proteins suggests dysregulation of the autophagy pathway (Fig. 4A). In response to IR, GAA levels increased over time in shControl U87MG cells, while no further induction occurred in shPYGL U87MG cells (Fig. 4A).

The mitochondrial protein HSP60 and the mitochondrial channel protein VDAC1 were higher in shPYGL than in shControl U87MG cells and remained elevated after IR (Fig. 4B). Furthermore, following IR the shPYGL cells became flat, enlarged and had increased lysosomal content (Fig. 4C). These are both features of senescence, known to be induced by both IR and PYGL knockdown. Moreover, after IR, accumulation of elongated and fragmented mitochondria was more evident in shPYGL compared to shControl cells, indicating a more profound senescence-like phenotype (Fig. 4C). Defects in mitophagy were also evident with accumulated mitochondria detected in lysosomes (Fig. 4C).

### Downregulation of PYGL inhibited glycolysis and mitochondrial respiration rates in response to IR

**Fig. 5:**
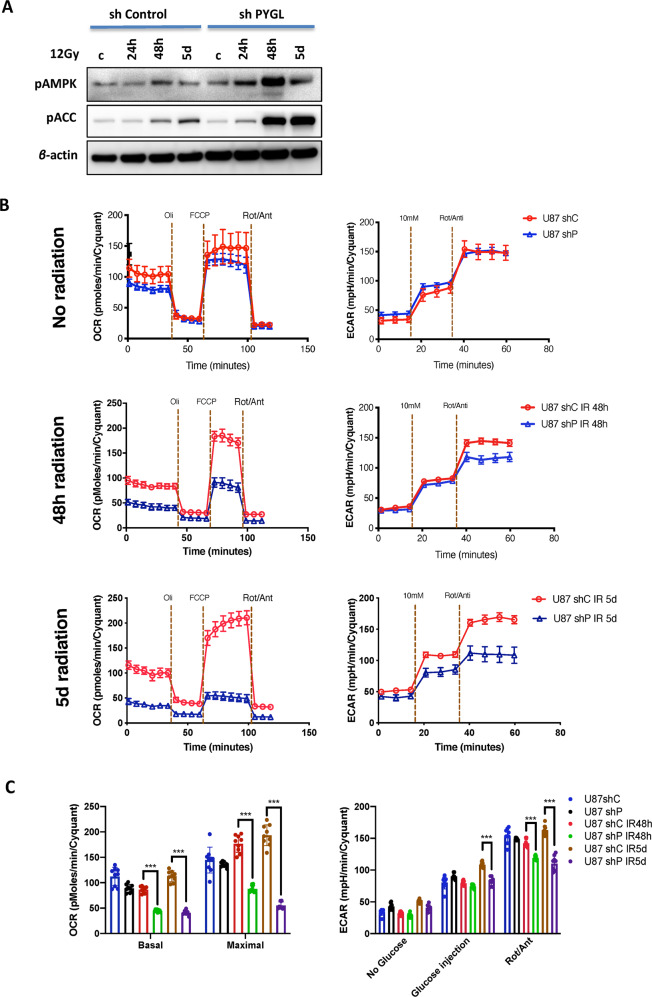
Inhibition of glycogen phosphorylase liver isoform (PYGL) alters the metabolic profile of glioblastoma (GBM) cell lines in response to ionising radiation (IR). **A** Western blot analysis of phosphorylated AMP-activated protein kinase (pAMPK) and its downstream target phosphorylated acetyl-CoA carboxylase (pACC) in control (shControl) and PYGL knockdown (shPYGL) U87MG cells 24 h, 48 h and 5 days after 12 Gy IR. **B**, **C** Basal and maximum oxygen consumption rate (OCR) and extracellular acidification rate (ECAR) are lower in shPYGL than shControl cells 48 h and 5 days after IR. (*n* = 3, error bars are ±SD, ***p < 0.001, *p* values were calculated by Two Way ANOVA - Tuckey post-hoc).

AMPK is an adenyl nucleotide sensor, activated by phosphorylation during states of low cellular energy levels. Protein expression levels of pAMPK and its downstream target pACC both increased 48 h and 5 days after IR and this was more pronounced in shPYGL cells than in shControl U87MG cells (Fig. 5A), suggesting energy stress in these cells. There was a reduction in both OCR and ECAR in shPYGL cells two days after IR, which further reduced 5 days after IR (Fig. 5B, C). These results suggest that degradation of glycogen is important for GBM cell survival after IR.

### Protein expression of enzymes involved in glycogen metabolism and their correlation with survival of patients with GBM

**Fig. 6:**
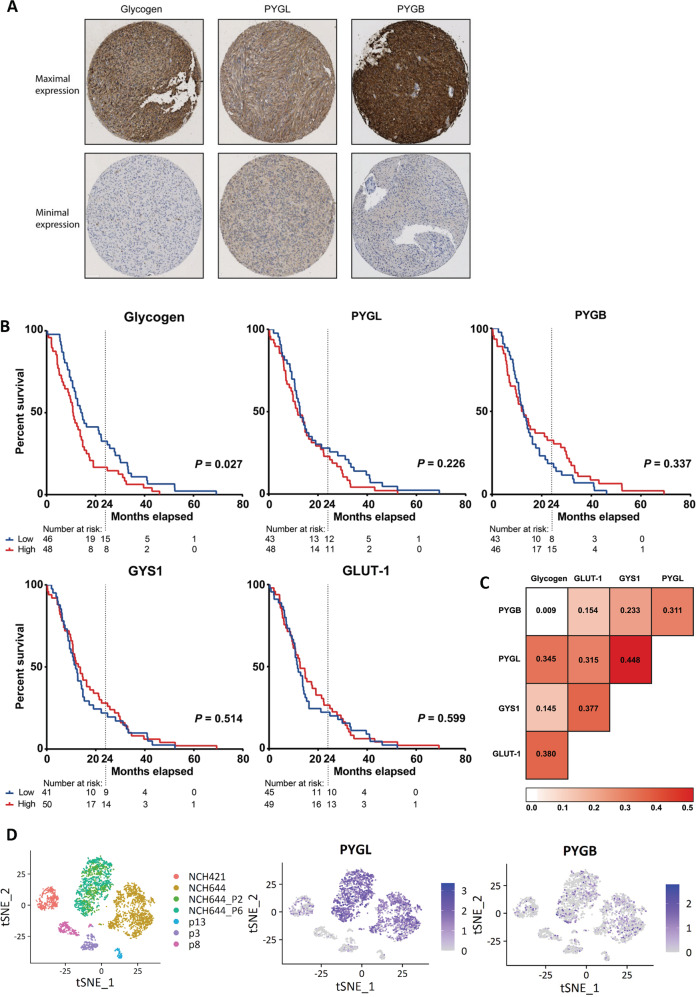
Gene and protein expression of glycogen and glycogen related enzymes in glioblastoma (GBM) patient tumours. **A** Maximal and minimal protein expression of glycogen, glycogen phosphorylase liver isoform (PYGL) and glycogen phosphorylase brain isoform (PYGB) observed in the tissue cores on the stained tissue microarray. **B** Kaplan-Meier curves plotted based on the mean protein expression score of three or four GBM tissue cores per patient younger than 70 years of age at date of surgery. Low or high protein expression of glycogen, PYGL and PYGB was determined based on the median score per patient in the complete cohort. **C** Heatmap showing Spearman’s correlations between protein expression scores of glycogen, glucose transporter 1 (GLUT-1), glycogen synthase 1(GYS1), PYGL and PYGB per glioblastoma tissue core. **D** t-Distributed Stochastic Neighbour Embedding (t-SNE) [67] plots showing the expression of PYGL and PYGB in FACS-sorted tumor cells of 3 GBM PDXs (P3, P8, P13) and 2 patient derived GBM cultures (NCH644, NCH421k) and subpopulations of NCH644 P2 (CD133 + CD44-A2B5 + CD15 + ) and P6 (CD133 + CD44 + A2B5 + CD15 + ). One sample per tumour is available.

Glycogen, GYS1, PYGL, PYGB and GLUT-1 proteins were all expressed in GBM cells as was confirmed by IHC on full tissue slides containing both normal brain and GBM tissue (Fig. S5B). There were very low glycogen, GYS1, PYGL and GLUT-1 expression levels, and low to median PYGB expression levels in healthy white matter and cortex tissue compared to GBM tissue. Numbers of patients per staining with sufficient evaluable cores are shown in Table S4, patient characteristics in Table S5. A large heterogeneity both between and within patients was found for immunohistochemical glycogen, GYS1, PYGL, PYGB and GLUT-1 expression on the TMA (Figs. 6A, S5C, D).

Univariate and multivariate Cox regression analyses showed age to be independently associated with overall survival (Table S6). Patients with low glycogen levels had better survival rates than patients with high glycogen levels (Fig. 6B). PYGL, PYGB, GYS1 and GLUT-1 expression levels did not influence survival rates (Fig. 6B).

GLUT-1 expression was positively correlated to glycogen, GYS1 and PYGL expression (Fig. 6C). This is in accordance with the glycogen accumulation and increased expression levels of GYS1 and PYGL we found in hypoxia in in vitro experiments. PYGL and GYS1 expression had the strongest positive correlation. PYGL expression, but not GYS1 expression, was correlated to glycogen expression.

Re-analyses of the single-cell sequencing of patient-derived xenografts and GBM stem-like cultures of Dirkse et al. [28, 29] showed low PYGL expression in the xenograft derived cells and higher expression in cells from the stem-like cultures, indicating that PYGL expression may be associated with stem-like features (Fig. 6D).

### Increased PYGL gene expression is related to hypoxia and associated with worse prognosis in patients with GBM

Consensus-ICA on the GEO dataset containing 4322 patient derived CNS samples including GBM, other CNS tumours and non-cancerous CNS tissues (Table S2) showed there were 169 TCs. Further, analysis shows that PYGL gene expression is positively associated with hypoxia and negatively with the TCA cycle and OXPHOS in GBM tissue and to a lesser extent in normal CNS tissue (Table S7, S8, S9 and Fig. S7A).

In the TCGA GBM data, PYGL and GYS1 mRNA expression levels were both higher in GBM tissue (*N* = 518) compared to normal brain tissue (*N* = 10) (Fig. S7B). Worse survival rates were seen in patients with high PYGL or high CA9 mRNA expression (Fig. S7C). The glycogen phosphorylase brain isoform, PYGB, was expressed at similar levels in both normal brains and glioblastoma tumors (Fig. S7B) and the mRNA expression levels did not correlate with survival rates (Fig. S7C). We furhter noted that there was a subset of tumours with high PYGM expression, although overall the tumour levels were lower than in normal brain (Fig. S7C). The survival in the top quartile (25%) of PYGM vs the remaining brain tumour samples did not differ (*p* = 0.09, data not shown) and did not correlate with high expression of PYGL (data not shown). PYGL and GYS1 mRNA expression levels were more strongly correlated than PYGB and GYS1 mRNA expression levels (Fig. S7D). Carbonic anhydrase 9 (CA9) was used as a marker of hypoxia. CA9 was more strongly correlated with GYS1 and PYGL mRNA expression levels than with PYGB mRNA expression levels (Fig. S7D). Single cell RNA sequencing from five primary gliomas [29, 30] was re-analysed and also showed PYGL expression clustering together with a hypoxia metagene (Fig. S7E) and glycolysis. PYGL, GBE1 and GYS1 are also aligned with each other and the hypoxia metagene, but PYGB showed a much wider spread.

## Discussion

In this study, we investigated the role of glycogen metabolism in the response of GBM cell lines to high dose lR. We found that inhibition of glycogen degradation by downregulation of PYGL, sensitised GBM cells to high dose IR.

In GBM, we would have expected a key role for the brain isoform PYGB. However, it was the liver isoform PYGL that was found to be upregulated in human GBM compared to normal brain. While both isoforms, PYGL and PYGB, are expressed in GBM cell lines, it was only the PYGL knockdown that inhibited growth of GBM cells. Also, the PYGM isoform was expressed in normal brain, while it was downregulated in glioblastoma patients. The GBM cell line U87MG was most sensitive to PYGL inhibition, indicating that highly metabolically active cells may be especially sensitive.

The glycogen phosphorylase isoforms are tightly regulated through both the binding of allosteric effectors and the phosphorylation of Ser14, in response to intracellular and extracellular energy demand, respectively. Phosphorylation of PYGL results in its full activation, allowing the use of liver glycogen in response to hypo- and hyperglycemic hormones but not via AMP [31]. On the other hand, PYGM strongly and cooperatively responds to AMP activation and to phosphorylation to control local energy needs while PYGB is non-cooperatively activated by AMP, and not by phosphorylation [31–36].

PYGB is important in some cancer cell lines such as pancreatic [37], prostate, ovary and gastric cancer cells [38–40], and for wound healing, invasion and metastasis in breast cancer cell lines [41]. Inhibition of PYGL induced cell death in hepatocellular carcinoma cell and potentiated the effects of multikinase inhibitors [42]. An additional factor, that may explain the lack of effect of PYGB knockdown in determining response to IR, is the recently reported redox switch that can specifically inactivate PYGB but not PYGL during oxidative stress [43].

At single standard radiotherapy doses of 4 to 8 Gy, the response in PYGL knockdown cells did not differ from control cells. However, at higher doses of IR or fractionated lower doses, there was a marked sensitisation after PYGL knockdown in GBM cell lines. Other strategies targeting glucose metabolism in GBM have previously been demonstrated to increase IR sensitivity in preclinical models [44, 45]. Live imaging indeed shows that high dose IR results in aberrant mitosis, mitotic catastrophe and the formation of giant multinuclear cells with senescence like phenotype, as demonstrated previously [46–48]. This phenotype was more pronounced in PYGL knockdown cells. It is well known that both IR and senescence also induce dysfunctional and elongated mitochondria [49, 50]. Conversely, cellular senescence is induced via deregulation of mitochondrial metabolism increased ROS, mitochondrial biogenesis, decreased mitophagy and fission [51].

Glycogen can be transported to autophagosomes via a selective process through starch-binding domain-containing protein 1 and GABARAP family protein interactions [52] and degraded in lysosomes via GAA [53, 54]. PYGL knockdown increased the basal levels of GAA and the lipidated forms of the autophagy markers GABARAPL1 and GABARAPL2, suggesting a compensatory mechanism of glycogen degradation via autophagy. In response to IR, the levels of GABARAPL1-II and GABARAPL2-II further accumulated over time in PYGL knockdown cells, while the levels of GAA remain unchanged. P62, which recognizes poly-ubiquitinated and un-ubiquitinated cargoes and transfers them to the autophagosomes for degradation [55, 56], was increased at baseline in PYGL knockdown cells and further accumulated in response to IR. High doses of IR are known to induce dysfunction of autophagy, accumulation of autophagosomes and lysosomal compartments, and p62 levels [57, 58]. The increased levels of p62 in shPYGL cells at baseline and in response IR might be related to reactive oxygen species, dysfunction of autophagy and development of senescence [59]. These effects appear to be enhanced by PYGL knockdown.

The dynamics of ATP response to glucose were slower in the PYGL knockdown cells than in control cells. There was a more rapid and higher induction of pAMPK and its substrate pACC in the PYGL knockdown cells, starting 24 h after IR. AMPK regulates metabolic energy needs and survival in response to IR, where activation of AMPK was found to switch the metabolic profile to mainly aerobic/anaerobic glycolysis [41, 60, 61]. Seahorse analysis showed a marked reduction in both oxidative phosphorylation and acid production in PYGL knockdown after IR. Thus insuficient glycogen degradation may result in inhibition of both glycolysis and TCA cycle activity resulting in reduced ATP production and subsequently an energy crisis in PYGLknockdown cells potentially preventing recovery from IR.

To assess the potential clinical relevance of PYGL and glycogen, we studied publicly available GBM RNA expression data sets and a well characterised series of GBM patients with TMA samples available. The importance of hypoxia as a regulator of PYGL expression was confirmed in the analysis of the GEO dataset. For the TMA, non-necrotic tumour areas in grade IV GBMs were selected. However GLUT-1 expression demonstrated that hypoxia was still present in the selected cores. In the TCGA dataset, high PYGL and CA9 mRNA expression were correlated with worse survival rates. This was not found for protein expression on the TMA, potentially due to the exclusion of necrotic tumour areas. Recently a similar analysis of the TCGA database also showed that PYGL was prognostic [62]. The higher expression we found of the PYGL compared to PYGB in GBM stem-like cultures suggests a role in plasticity and tumorigenesis [28]. Although glycogen metabolism is an emerging target for therapy and PYGL inhibitors have been clinically tested up to phase II studies, results have not been published so far [63–66].

**Fig. 7:**
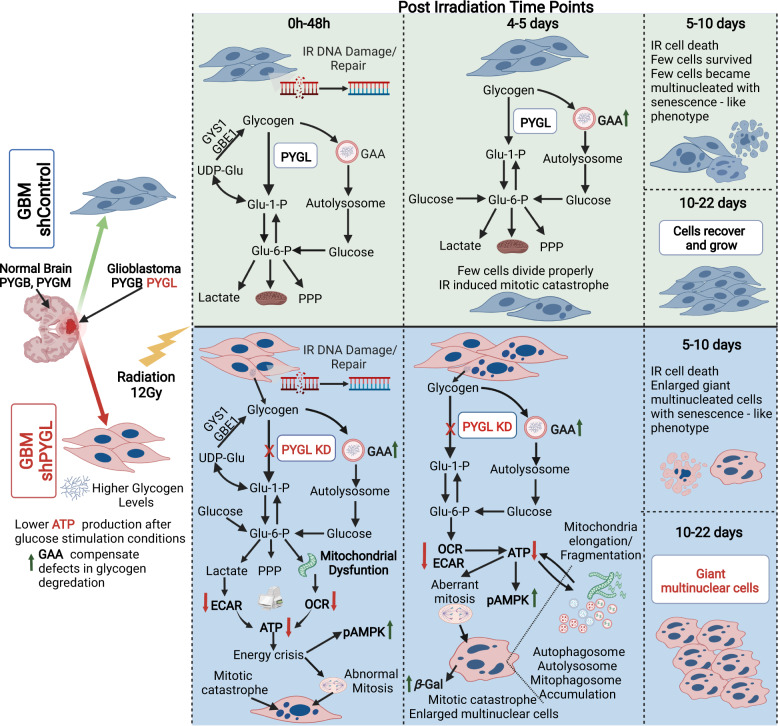
Schematic time-course changes in cellular processes in phosphorylase liver isoform knockdown (shPYGL) and control (shControl) U87MG glioblastoma (GBM) cells following high dose ionising radiation (IR). PYGB Glycogen phosphorylase brain isoform, PYGM Glycogen phosphorylase muscle isoform, PYGL Glycogen phosphorylase liver isoform, GAA alpha-acid glucosidase, GYS1 Glycogen Synthase 1, GBE1 Glycogen branching enzyme 1, AMPK AMP-activated protein kinase, *β*-gal *β*-galactosidase, PPP Pentose phosphate pathway, OCR Oxygen consumption rate, ECAR Extracellular acidification rate. (Figure created with BioRender.com).

We propose that accumulation of glycogen due to PYGL knockdown results in lysosomal stress and impaired autophagy and mitophagy after IR (Fig. 7). The resulting metabolic deficiencies affect the availability of ATP required for mitosis, resulting in aberrant mitosis and development of mitotic catastrophe and giant multinuclear cells with senescence like phenotype following IR (Fig. 7). Radiotherapy is a cornerstone of GBM treatment, and we have shown the substantial effects of PYGL knockdown in enhancing radiation induced cell killing. This indicates that PYGL is a potential target to increase the radiosensitivity of GBM.

### Reporting summary

Further information on research design is available in the Nature Research Reporting Summary linked to this article.

## Supplementary information

Original Data File

SUPPLEMMEENTAL MATERIAL METHODS

Figure S1

Figure S2

Figure S3

Figure S4

Figure S5

Figure S6

Figure S7

shControl U87MG no radiation

shControl with radiation and mitotic catastrophe

shControl with radiation no mitotic catastrophe

shPYGL U87MG no radiation

shPYGL with radiation and mitotic catastrophe

Table 7S

Table S8

Table 9S

Reporting Summary

## Data Availability

The codes used to support tumour mRNA transcript analysis will be made available upon request.

## Data availability

The TMAs are available for collaboration on discussion with the authors
