# Supplementary material for: Liver glycogen phosphorylase is upregulated in glioblastoma and provides a metabolic vulnerability to high dose radiation
Source: Cell Death Dis. 2022 Jun 28;13(6):573. doi: 10.1038/s41419-022-05005-2 (PMC9240045; doi:10.1038/s41419-022-05005-2)
Supplement: Supplementary file 1 — Original Data File [file 41419_2022_5005_MOESM1_ESM.pptx]

## Slide 1
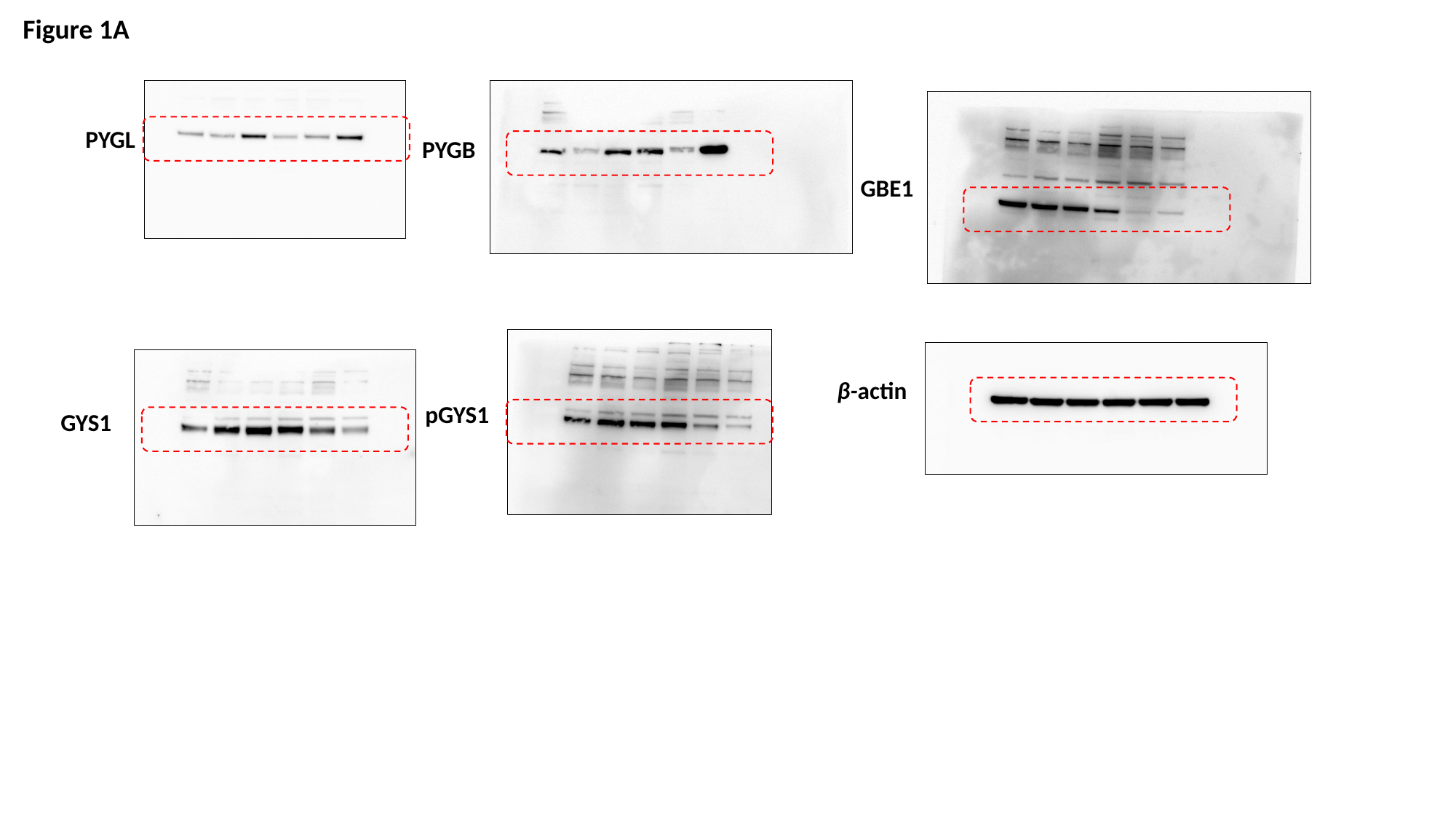

Figure 1A
PYGL
PYGB
GBE1
β-actin
pGYS1
GYS1

## Slide 2
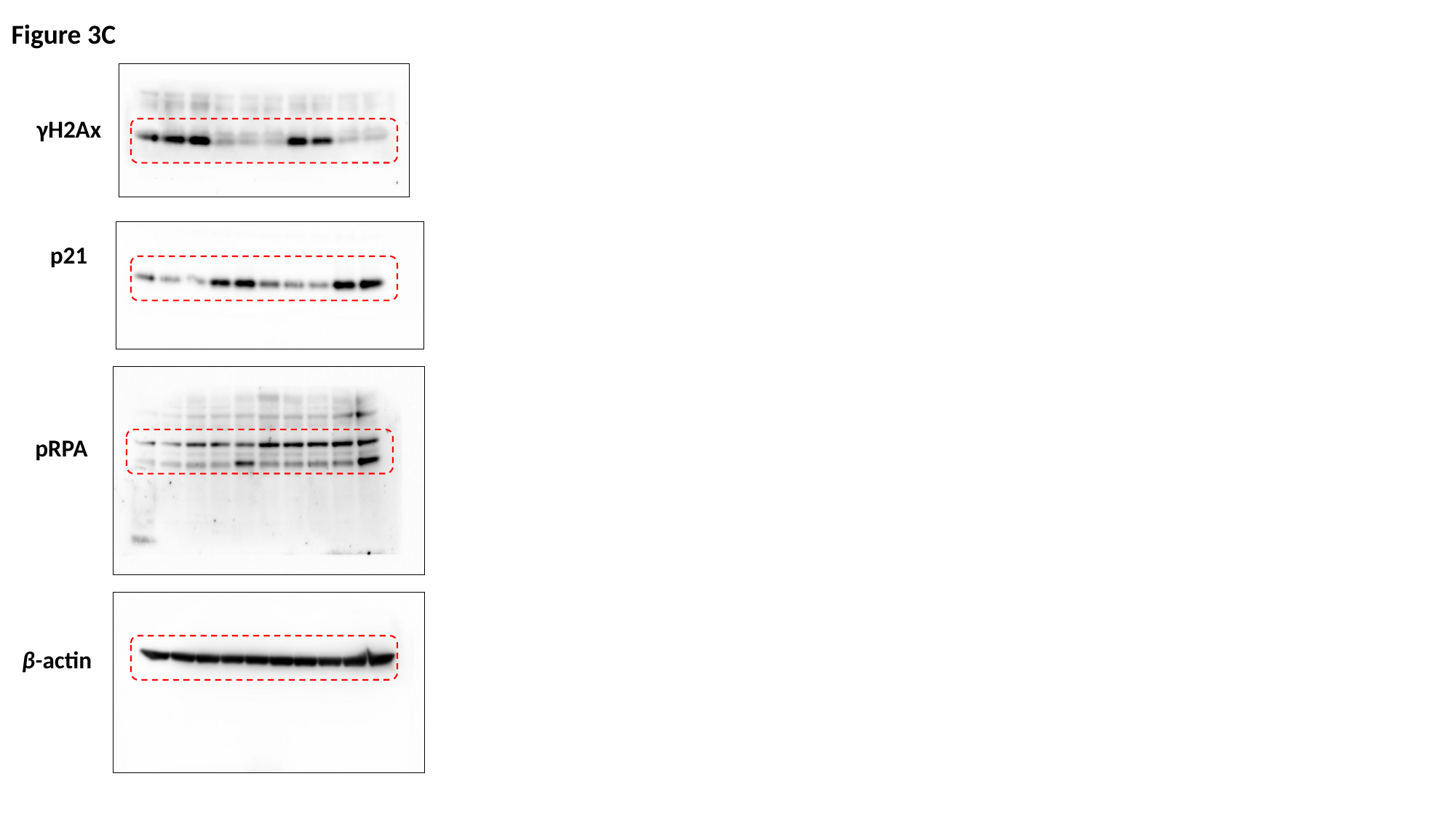

Figure 3C
γH2Ax
p21
pRPA
β-actin

## Slide 3
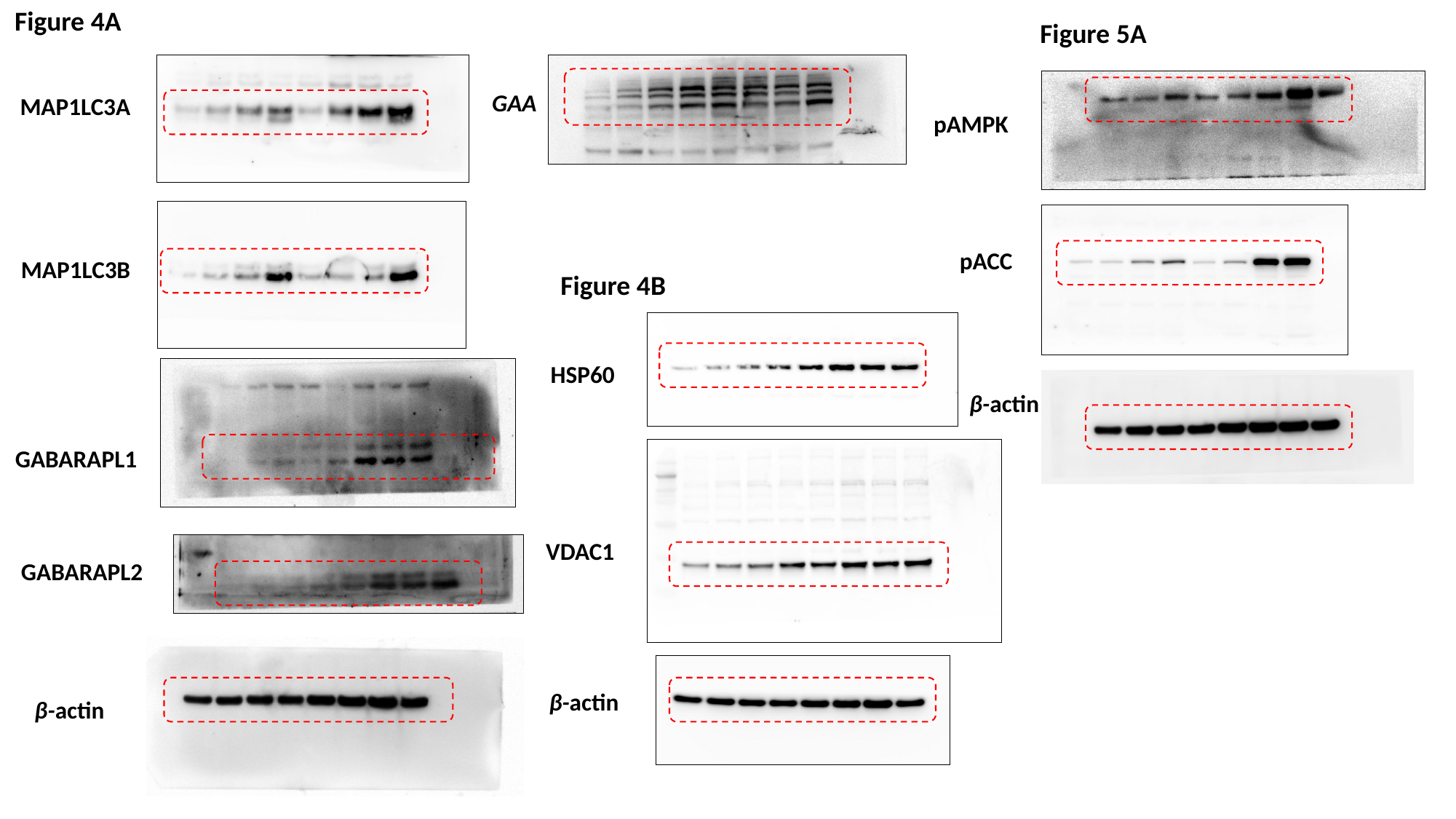

Figure 4A
Figure 5A
GAA
MAP1LC3A
pAMPK
pACC
MAP1LC3B
Figure 4B
HSP60
β-actin
GABARAPL1
VDAC1
GABARAPL2
β-actin
β-actin
