# Supplementary material for: Liver glycogen phosphorylase is upregulated in glioblastoma and provides a metabolic vulnerability to high dose radiation
Source: Cell Death Dis. 2022 Jun 28;13(6):573. doi: 10.1038/s41419-022-05005-2 (PMC9240045; doi:10.1038/s41419-022-05005-2)
Supplement: Supplementary file 2 — SUPPLEMMEENTAL MATERIAL METHODS [file 41419_2022_5005_MOESM2_ESM.docx]

**SUPPLEMENTAL METHODS**

*Immunohistochemistry*

Antigen retrieval of deparaffinised GBM tissues was conducted using Tris/HCl buffer with pH 9.0. Endogenous peroxidase was blocked by incubating in 0.3% H_2_O_2_ for 30 min at RT. Tissue sections were incubated at RT for 1 h with a primary antibody to glycogen (gift from professor O. Baba ([24](#_ENREF_24))), GYS1 (Abcam), PYGL (Atlas), PYGB (Atlas), all 1:200 dilution; GLUT-1 (Millipore) 1:750 dilution). This was followed by 30 min incubation at RT with respectively a secondary antibody (peroxidase-conjugated, DAKO) and a tertiary antibody (peroxidase-conjugated, DAKO), each diluted 1:100 in 1% bovine serum albumin (BSA)/PBS + 1% AB serum. Colour development was obtained by incubation with 3,3’-diaminobenzidine (DAB) diluted in PBS and hydrogen peroxide for 10 min. Haematoxylin was used for counterstaining, followed by mounting. Slides were digitalised using C9600 NanoZoomer (Hamamatsu Photonics KK, Hamamatsu City, Japan).

The algorithm incorrectly scored dark blue nuclei as being brown staining, which can be considered as a standard error. To prove this, 20 biopsies selected at random per staining were scored manually according to the following formula: H-score = (1 x percentage of biopsy moderate staining) + (2 x percentage of biopsy high staining). The automatically and manually defined H-scores of these 20 biopsies per staining did not differ as tested by the Wilcoxon signed-rank test. In normal brain, glioma and kidney tissue samples, digital scoring using Aperio’s positive pixel count algorithm has been proven to strongly correlate to manual scoring and exceed the reproducibility of manual scoring ([68](#_ENREF_68), [69](#_ENREF_69)).

**Figure S1.**

A. Pan-cancer analysis of glycogen phosphorylase liver isoform (PYGL) gene expression, examined using Gene Expression Profiling Interactive Analysis ([27](#_ENREF_27)) in normal (N, green) and tumour (T, red) tissues. B. Expression levels of all glycogen phosphorylase isoforms (PYGL, PYGB, PYGM) in normal brain (N=207) and glioblastoma patients (N=163). C. Heatmap analysis of those isoforms in different normal brain regions, using the Gene Expression Profiling Interactive Analysis tool (GEPIA, ([27](#_ENREF_27))). D. Validation of the siRNAs against PYGL and PYGB in U87MG, U251MG, U118MG, LN229, LN18 and T98G. Different exposure times for the western blots in each cell line were used to highlight the level of the knockdown.

**Figure S2. Bioenergetic profiles and glycogen production in glioblastoma cell lines.**

**A.** Quantification of the basal oxygen consumption rate (OCR) taken from Figure 1C as the average of the three measurement and indicates the baseline of respiration of the cells in the presence of 5mM glucose (n=3, error bars are ±SD).

**B.** Quantification of the Maximal OCR was taken from Figure 1C as the average of the three measurements after the FCCP injection at 5mM glucose (n=3, error bars are ±SD).

**C.** Quantification of the Extracellular acidification rate (ECAR) taken from the Figure 1D as the average of the three measurements after 10mM glucose injection (n=3, error bars are ±SD).

**D.** Quantification of the maximal ECAR taken from Figure 1D as the overage of the three measurements after the injection of rotenone and antimycin A in 10mM glucose (n=3, error bars are ±SD).

**E.** Fold changes in glycogen levels in normoxia (21% oxygen) compared to hypoxia (0.1% oxygen) in 6 GBM cell lines (n=3, error bars are ±SD).

**Figure S3.**

**A,B.** Cell numbers in response to 6, 8 and 10 Gy in shControl and shPYGL of U251MG and T98G cell lines at 15 days (n=3, Error bars are ± SD, **p<0.01, ***p<0.001, p values were calculated by unpaired t-test). Due to differing sensitivities to irradiation, the radiation doses for the U251MG cell line are lower than for the other cell lines.

**C.** Western blot validation of the efficacy of short hairpin RNAs against PYGL and PYGB in the U87MG cell line in the time course post-IR.

**Figure S4.**

Time lapse video of shControl and shPYGL U87MG cells with or without ionising radiation at 72-96h time interval. i) time lapse video of shControl U87MG cells. ii) time lapse video of shPYGL U87MG cells. iii) Time lapse video of shControl U87MG cells following IR, where a cell can divide without any visible defects. iv) time lapse video of shControl U87MG cells following IR, where the cell undergoes mitotic catastrophe. v) Time lapse video of shPYGL U87MG cells, where the cell undergoes mitotic catastrophe.

**Figure S5.**

**A.** Sequential full slides containing normal brain and grade IV glioblastoma (GBM) tissue. Top left panel shows annotation of the tumour areas: most normal neocortex (dark blue), infiltration zone of the cortex (light blue), vital tumour areas (red) and necrotic areas (yellow). Tissue slides immunohistochemically stained for phosphorylase liver isoform (PYGL), glycogen phosphorylase brain isoform (PYGB), glycogen, glycogen synthase 1 (GYS1), glycogen and glucose transporter 1 (GLUT-1). **B.** CONSORT diagram showing patient selection from the available tissue microarray (TMA). **C.** Immunohistochemical staining of grade IV glioblastoma tissue on the TMA for glycogen, GYS1, PYGL, PYGB and GLUT-1. These are sequential cores of three different patients showing interpatient heterogeneity. **D.** Immunohistochemical staining of grade IV glioblastoma tissue on the TMA for glycogen, GYS1, PYGL, PYGB and GLUT-1. For each staining, this Figure includes three tissue cores of one patient showing intrapatient heterogeneity.

**Figure S6.**

Histograms showing the distribution of average expression scores based on at least three tissue cores per patient for glycogen (*N*_patients_= 123), GYS1 (*N*_patients_= 120), PYGL (*N*_patients_= 119), PYGB (*N*_patients_= 116) and GLUT-1 (*N*_patients_= 123) expression. Medians are shown as red lines.

**Figure S7.**

**A.** Violin plots showing the activity scores of transcriptional components (TC) 19 and 63 within normal brain tissue (*N* = 82), post-mortem normal brain tissue (*N* = 19) and glioblastoma tissue (*N* = 797). TC19 has a higher activity in GBM tissue compared to normal brain tissue obtained when patients were alive (*N*=82) and post-mortem (*N*=19). TC19 captures the regulatory process most positively related to the gene expression of PYGL and coregulated genes. TC63 captures the regulatory process most negatively related to the gene expression of PYGL and coregulated genes. TC63 has a lower activity in GBM tissue compared to normal brain tissue when patients were alive (*N*=82) and post-mortem (*N*=19).

**B.** Box plots showing mRNA abundance of the glycogen phosphorylase isoforms (PYGL, PYGB, PYGM) and glycogen synthase (GYS1, GYS2) across glioblastoma patients (blue, T) and normal brain samples (white, N) based on TCGA data. The horizontal grey line is drawn at the highest expression value of GYS2, which is our control to highlight the threshold of low gene expression in the brain. The vertical grey line is a separator between the control (GYS2) and other genes.

**C.** Kaplan Meier survival analysis was performed on the TCGA glioblastoma dataset. Cox proportional hazard model was fit to median dichotomised mRNA abundance and P values were estimated using Wald test.

**D.** mRNA abundance correlation heatmap based on TCGA glioblastoma cohort. Numbers and intensity represent Spearman’s correlation coefficient.

**E.** Re-analysis of single cell data from 5 human gliomas (colour coded MGH 26-31) ([30](#_ENREF_30)). Heatmap shows individual cells from the 5 tumours, top line, and their expression levels for indicated genes and pathways on the vertical axis.

**SUPPLEMENTAL TABLES**

**Table S1. Characteristics of the glioblastoma cell lines U87MG, T98G, U118MG, U251MG, LN18, LN229.**

|  | U87MG | U118MG | U251MG | T98G | LN18 | LN229 |
| --- | --- | --- | --- | --- | --- | --- |
| Organism | human | human | human | human | human | human |
| Tissue | brain | brain | brain | brain | brain | brain |
| Disease | GBM | GBM | GBM | GBM | GBM | GBM |
| p53 | wt | wt | mutated | mutated | mutated | mutated |
| PTEN | mutated | wt | wt | mutated | wildtype | wildtype |
| IDH-1 | wt | wt | wt | wt | wt | wt |

GBM: glioblastoma. PTEN: phosphatase and tensin homolog. IDH-1: Isocitrate dehydrogenase-1.

**Table S2. Central nervous system samples included in Gene Expression Omnibus analysis.**

| Tissue type | Number of samples |
| --- | --- |
| Adamantinomatous craniopharyngioma | 24 |
| Anaplastic astrocytoma | 80 |
| Anaplastic carcinoma | 32 |
| Anaplastic oligoastrocytoma | 40 |
| Anaplastic oligodendroglioma | 32 |
| Anaplastic pleomorphic xanthoastrocytoma | 2 |
| Astrocytoma | 210 |
| Atypical teratoid / rhabdoid tumor | 79 |
| Brainstem glioma | 6 |
| Choroid plexus papilloma | 5 |
| Desmoplastic infantile ganglioglioma | 4 |
| Embryonal tumor with multilayered rosettes | 10 |
| Ependymoblastoma | 1 |
| Ependymoma | 394 |
| Ganglioglioma | 20 |
| Ganglioneuroblastoma | 2 |
| Glioblastoma | 797 |
| Glioma | 21 |
| Malignant peripheral nerve sheath | 6 |
| Medulloblastoma | 353 |
| Meningioma | 167 |
| Neuroblastoma | 286 |
| Central nervous system – metastasis | 8 |
| Central nervous system – normal histology | 82 |
| Central nervous system – not specified | 142 |
| Central nervous system – other disease | 401 |
| Central nervous system – vitro / cell | 5 |
| Central nervous system post-mortem – normal histology | 19 |
| Central nervous system post-mortem – not specified | 117 |
| Central nervous system post-mortem – other disease | 561 |
| Oligoastrocytoma | 18 |
| Oligodendroglioma | 84 |
| Pediatric high grade glioma | 2 |
| Pilocytic astrocytoma | 179 |
| Pineoblastoma | 1 |
| Pleiomorphic xanthoastrocytoma | 5 |
| Primitive neuroectodermal tumor | 82 |
| Schwann cells | 2 |
| Schwannoma | 1 |
| Primary central nervous system lymphoma | 42 |

**Table S3. The 16 gene set collections used for GSEA (**[**70-74**](#_ENREF_70)**).**

| Name of gene set collection | Number of gene sets |
| --- | --- |
| BioCarta | 289 |
| Cancer gene neighbourhoods | 427 |
| Cancer modules | 431 |
| Chemical and genetic perturbations | 3302 |
| Gene ontology – biological process | 7350 |
| Gene ontology – cellular component | 1001 |
| Gene ontology – molecular function | 1645 |
| Hallmark | 50 |
| Human phenotype ontology | 3100 |
| Immunologic signatures | 4872 |
| KEGG | 186 |
| Mammalian phenotypes | 4004 |
| MicroRNA targets | 221 |
| Oncogenic signatures | 189 |
| Reactome | 1499 |
| Transcription factor targets | 610 |

**Table S4. Numbers of grade IV glioblastoma patients of whom at least three evaluable tumour cores were available on the TMA and total numbers of evaluable tumour cores available on the TMA per staining.**

|  | Glycogen | GYS1 | PYGL | PYGB | GLUT-1 |
| --- | --- | --- | --- | --- | --- |
| Patients, *N* | 123 | 120 | 119 | 116 | 123 |
| Cores, *N* | 452 | 432 | 436 | 421 | 457 |

**Table S5. Patient characteristics of the evaluable patients on the TMA.**

| Number of patients, *N* | 123 |
| --- | --- |
| Age at surgery in years, *median (range)* | 63.8 (36.6 – 84.6) |
| Male, *N (%)* | 79 (64%) |
| Still alive, *N* | 0 |
| Overall survival in months, *median (range)* | 10.7 (0.2 – 69.3) |
| EGFR amplification (*N=120)*, *N (%)* | 58 (48%) |

Overall survival is the time between surgery and death. EGFR = epidermal growth factor receptor.

**Table S6. Univariate and multivariate Cox regression to determine factors associated with overall survival of patients with glioblastoma of whom tissue was included in the tissue microarray.**

|  | **Univariate analysis** | | **Multivariate analysis** | |
| --- | --- | --- | --- | --- |
| **Variable** | **HR (95% CI)** | ***P*-value** | **HR (95% CI)** | ***P*-value** |
| **Glycogen score** | 1.05 (0.97 - 1.13) | 0.236 | 1.04 (0.95 – 1.2) | 0.385 |
| **GYS1 score** | 0.98 (0.90 – 1.07) | 0.676 | 0.98 (0.89 – 1.08) | 0.715 |
| **PYGL score** | 1.04 (0.91 – 1.18) | 0.603 | 1.05 (0.89 – 1.25) | 0.564 |
| **PYGB score** | 0.98 (0.94 – 1.03) | 0.439 | 0.99 (0.94 – 1.04) | 0.567 |
| **GLUT-1 score** | 0.98 (0.93 – 1.03) | 0.369 | 0.97 (0.91 - 1.03) | 0.297 |
| **Age at surgery** | 1.04 (1.02 – 1.07) | **<0.001** | 1.04 (1.02 – 1.06) | **0.002** |
| **Female** | 0.96 (0.66 – 1.40) | 0.849 | 1.03 (0.69 – 1.55) | 0.872 |
| **EGFR amplification** | 0.73 (0.51 – 1.06) | 0.733 | 1.23 (0.83 – 1.84) | 0.304 |

Mean protein expression scores per patient were determined based on at least three evaluable tissue cores stained for glycogen (*N*_patients_= 123), glycogen synthase 1 (GYS1, *N*_patients_= 120), glycogen phosphorylase liver isoform (PYGL, *N*_patients_= 119), glycogen phosphorylase brain isoform (PYGB, *N*_patients_= 116) and GLUT-1 (*N*_patients_= 123). HR: hazard ratio; EGFR: epidermal growth factor receptor. Enter method was used for multivariate analysis.

**Table S7. Ranked weights of PYGL gene expression in all transcriptional components.**

PYGL gene expression had the highest ranked weight from the top in TC number 19 with a positive Z-score, indicating that this TC captures the regulatory process most positively related to the mRNA expression of PYGL and coregulated genes. PYGL gene expression has the highest ranked weight from the bottom in TC63 with a negative Z-score, indicating that this TC captures the regulatory process most negatively related to the gene expression of PYGL and coregulated genes.

**Table S8. Top 10 enriched gene sets with a Z-score ≥3 per gene set database in transcriptional component 19.**Hypoxia and glycolysis related gene sets are highlighted. GSEA with 16 gene set collections showed positive enrichment of TC19 for gene sets describing hypoxia and glycolysis according to multiple gene set collections, and for hypoxia-inducible factor (HIF) 1 transcription factor targets.

**Table S9. Top 10 enriched gene sets with a Z-score ≥3 per gene set database in transcriptional component 63.** Tricarboxylic acid cycle (TCA cycle) and oxidative phosphorylation (OXPHOS) related gene sets are highlighted. GSEA with 16 gene set collections showed positive enrichment of TC63 for gene sets describing the tricarboxylic acid cycle (TCA cycle) and oxidative phosphorylation (OXPHOS) according to multiple gene set databases (Table S9).
