# Supplementary figures and images for: Liver glycogen phosphorylase is upregulated in glioblastoma and provides a metabolic vulnerability to high dose radiation
Source: Cell Death Dis. 2022 Jun 28;13(6):573. doi: 10.1038/s41419-022-05005-2 (PMC9240045; doi:10.1038/s41419-022-05005-2)

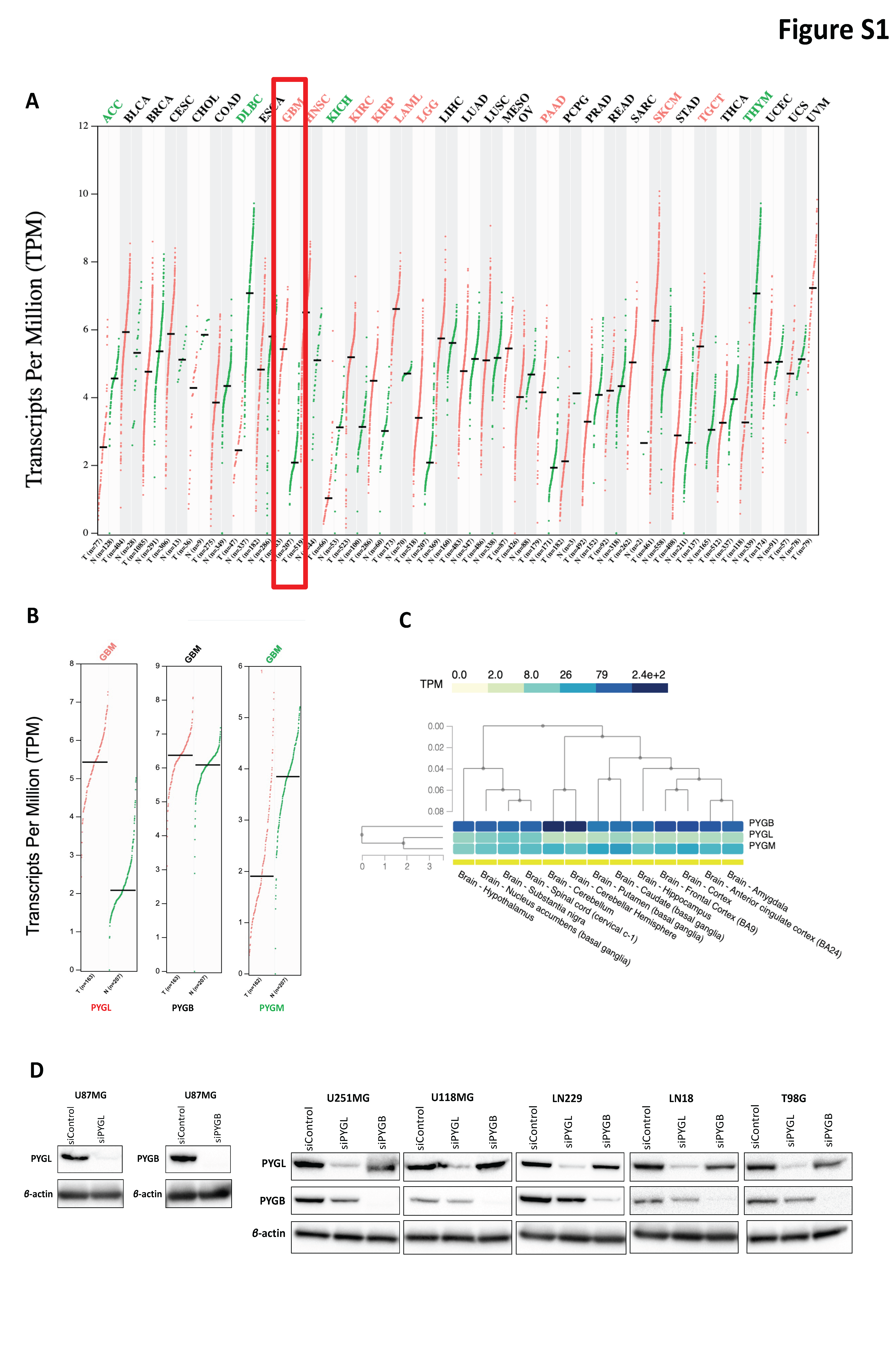

Supplement: Supplementary file 3 — Figure S1 [file 41419_2022_5005_MOESM3_ESM.tif]

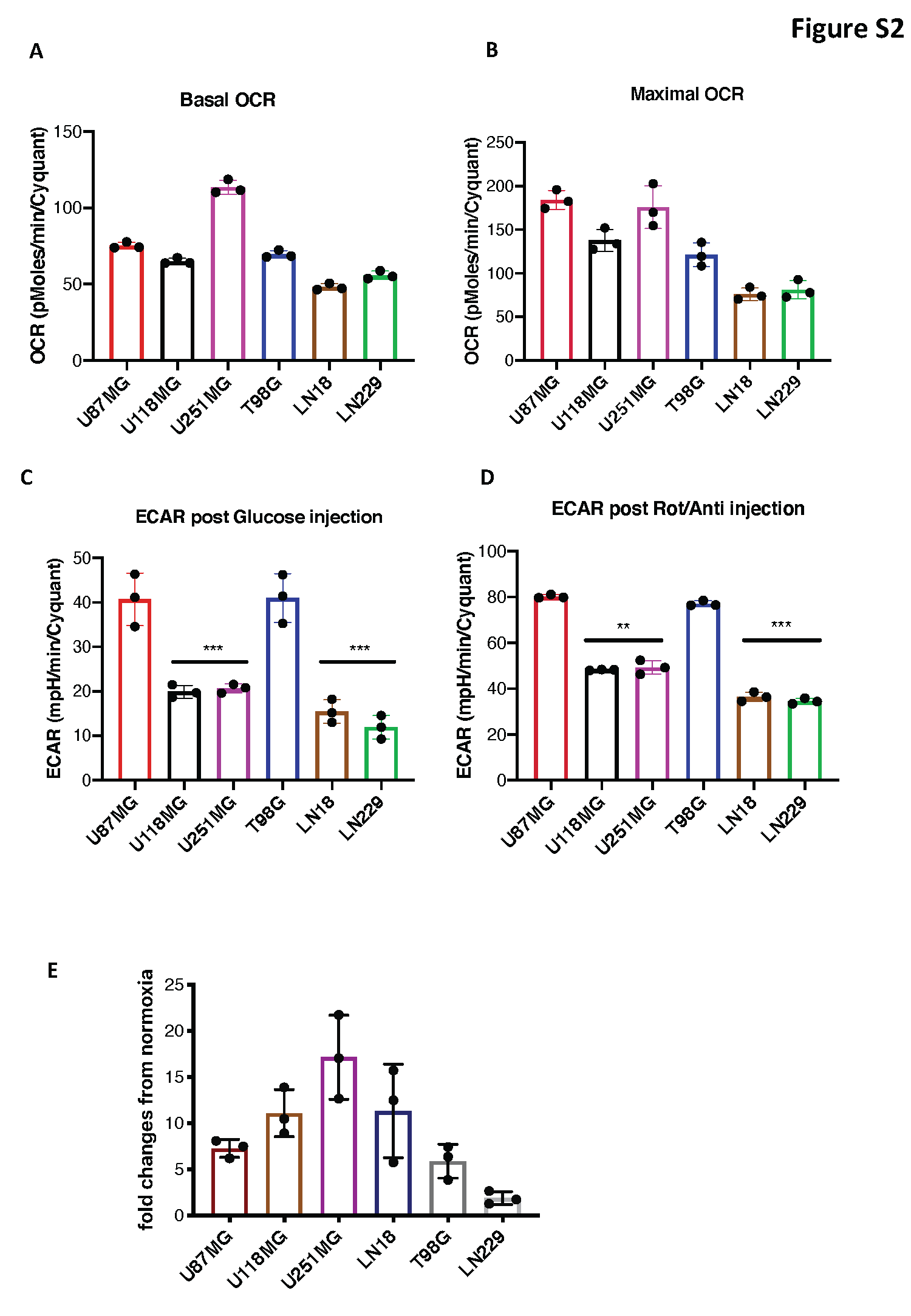

Supplement: Supplementary file 4 — Figure S2 [file 41419_2022_5005_MOESM4_ESM.tif]

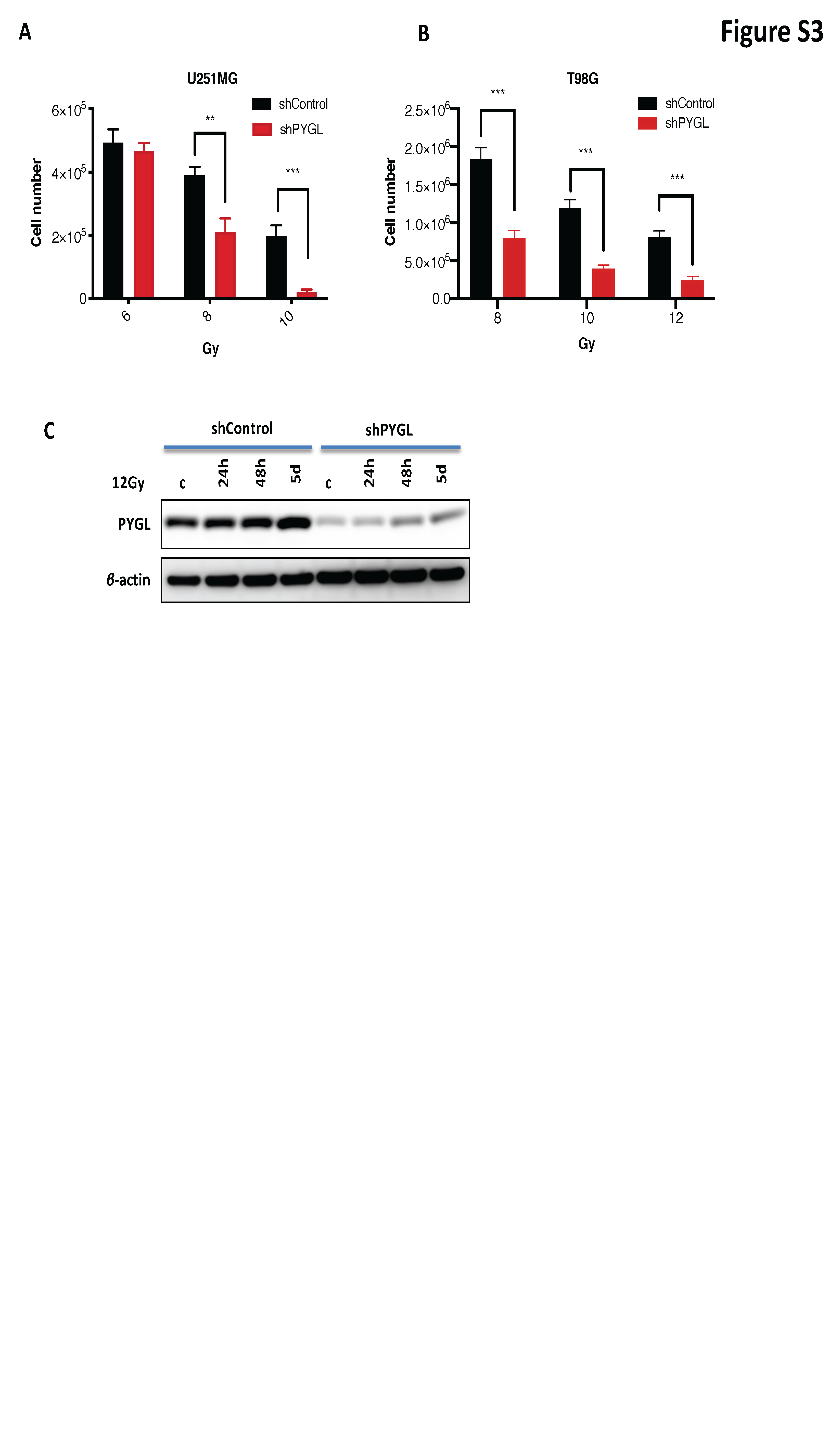

Supplement: Supplementary file 5 — Figure S3 [file 41419_2022_5005_MOESM5_ESM.tif]

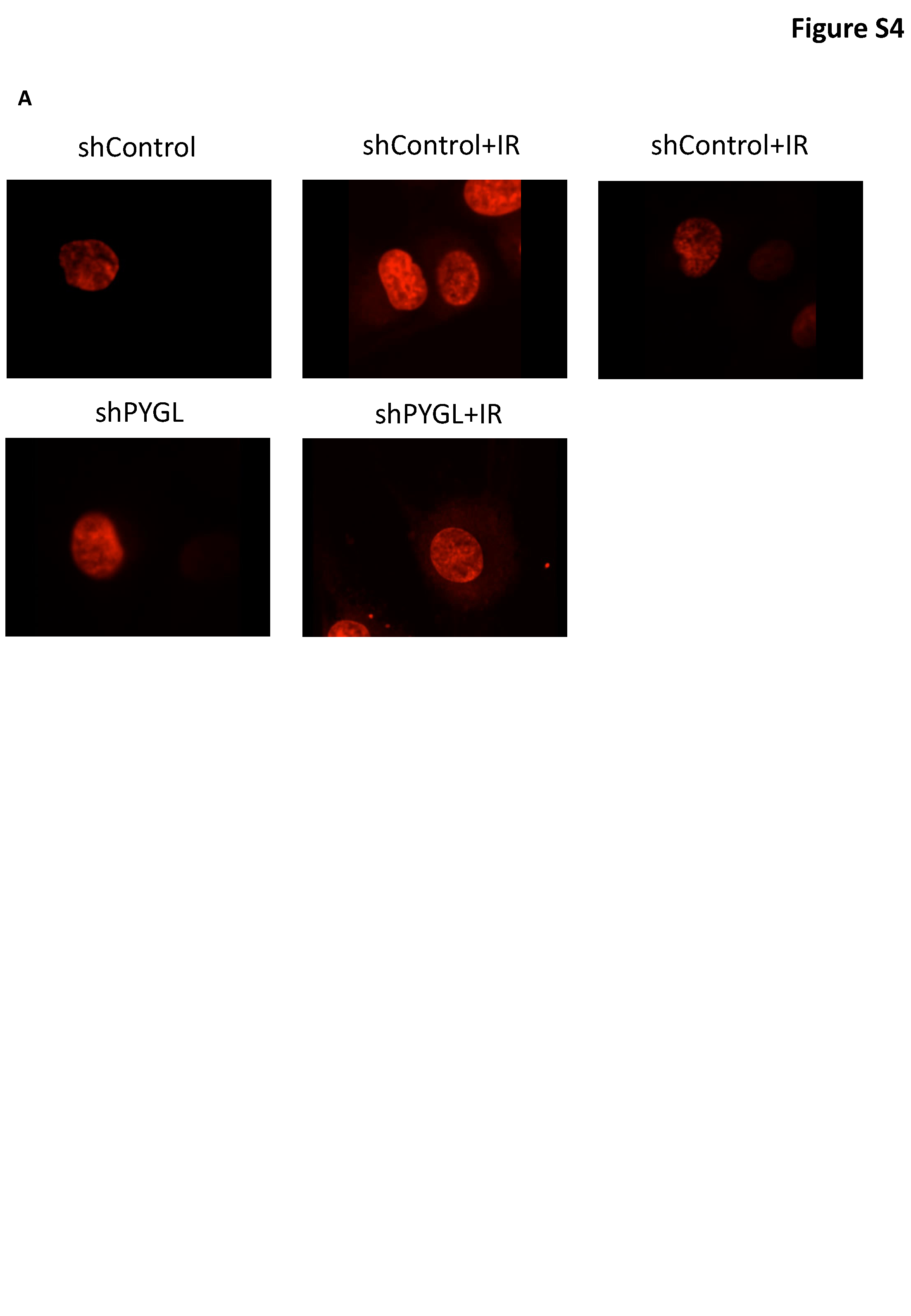

Supplement: Supplementary file 6 — Figure S4 [file 41419_2022_5005_MOESM6_ESM.tif]

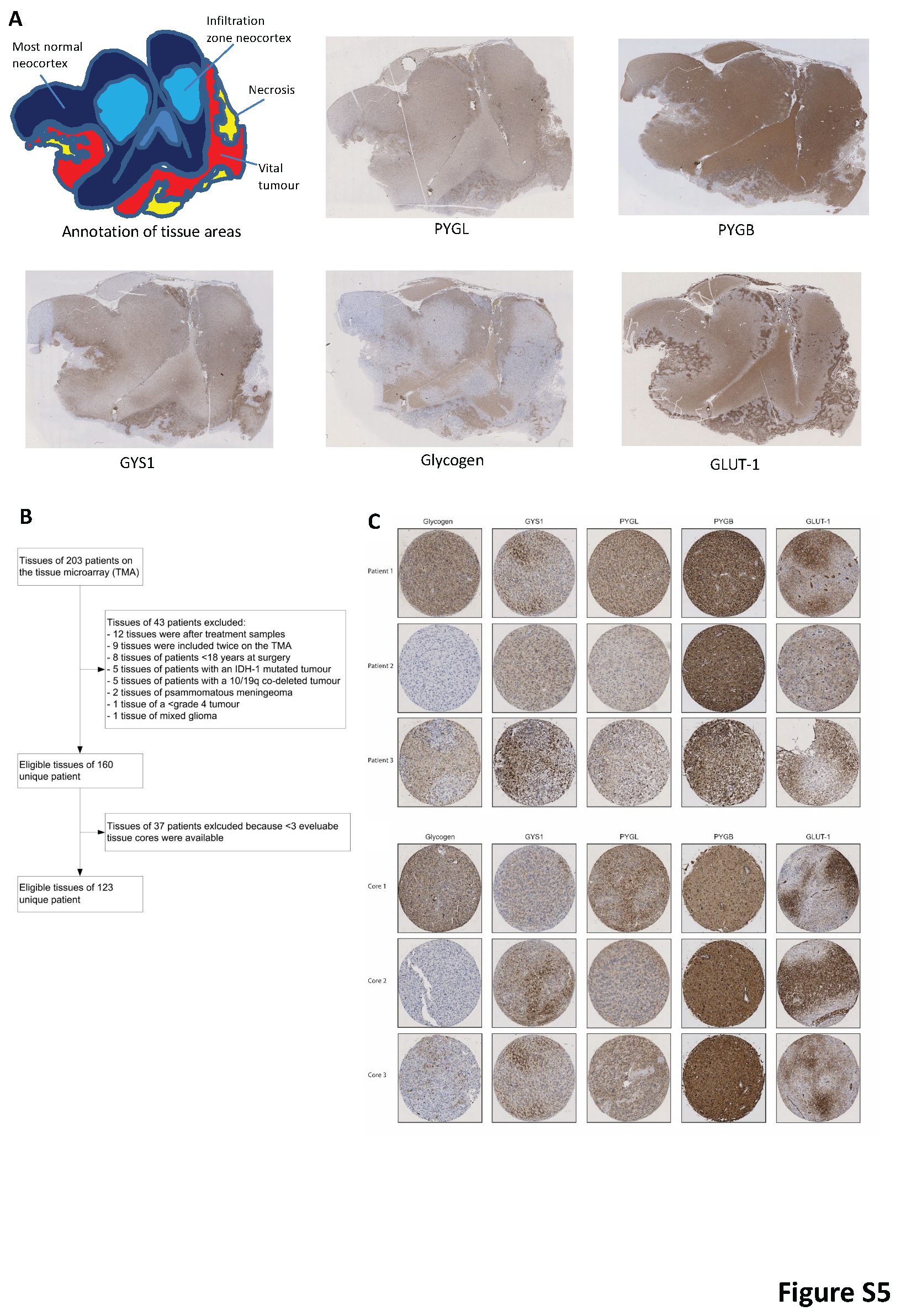

Supplement: Supplementary file 7 — Figure S5 [file 41419_2022_5005_MOESM7_ESM.tif]

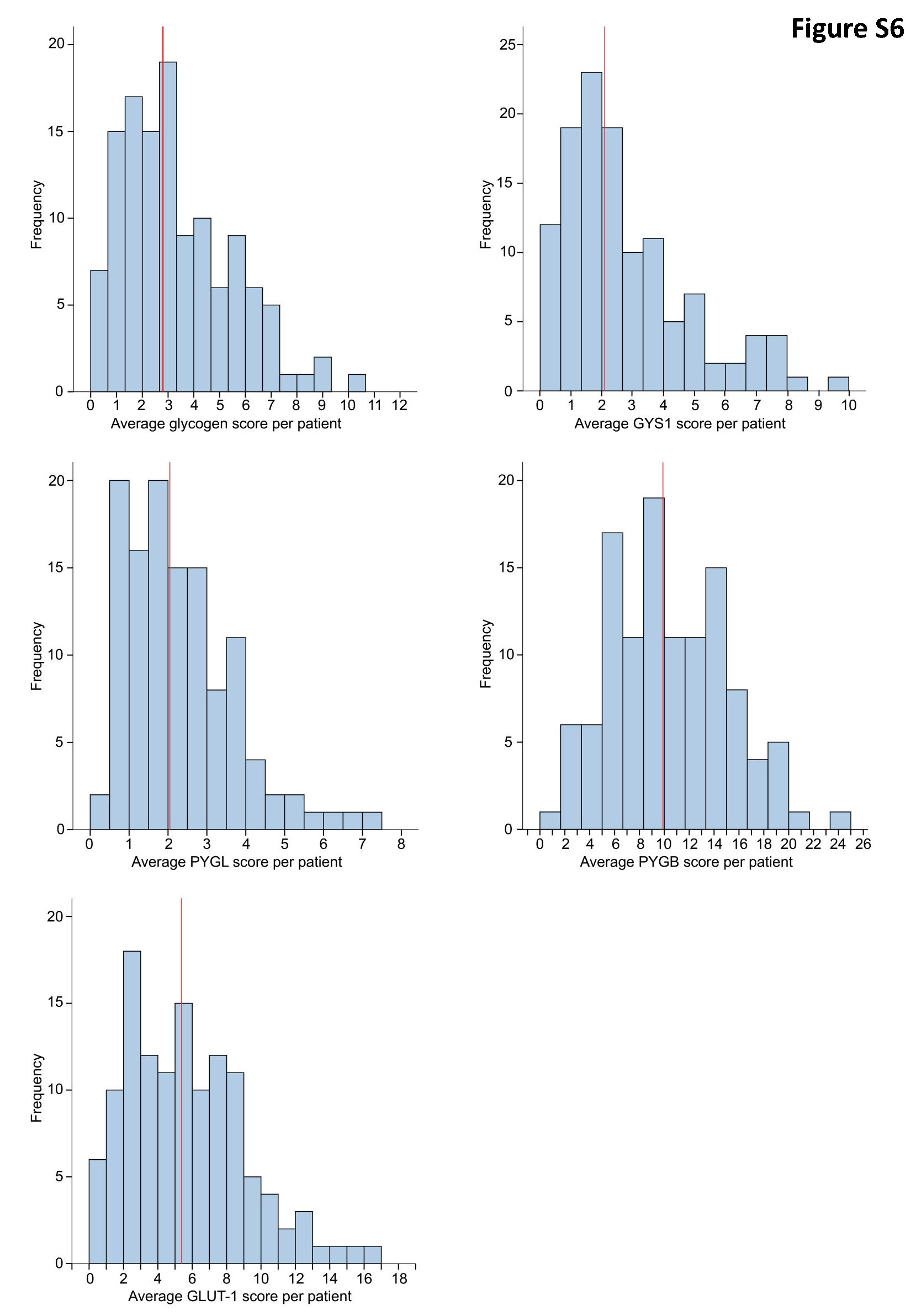

Supplement: Supplementary file 8 — Figure S6 [file 41419_2022_5005_MOESM8_ESM.tif]

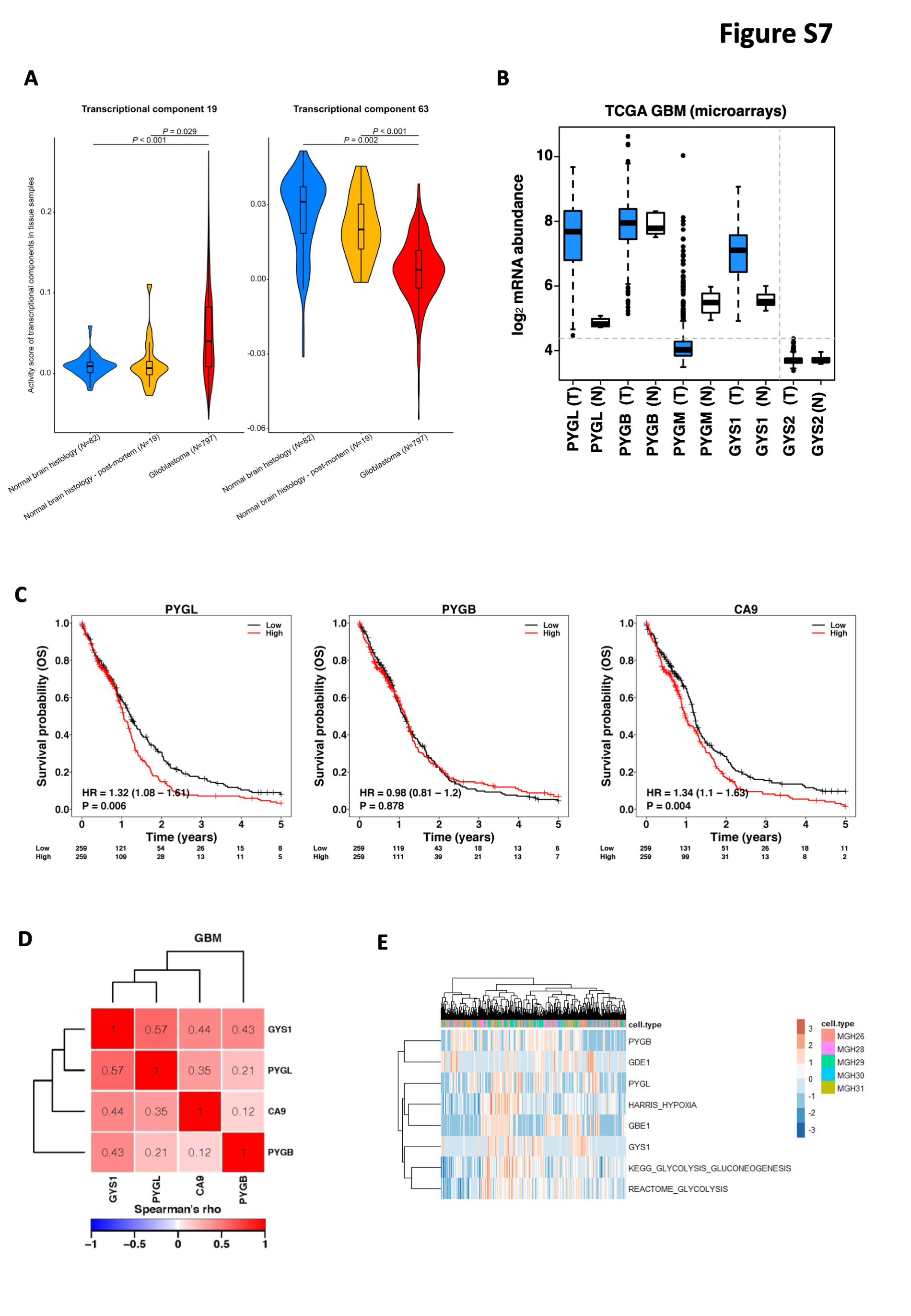

Supplement: Supplementary file 9 — Figure S7 [file 41419_2022_5005_MOESM9_ESM.tif]
